# A Structure-Based Mechanism for DNA Entry into the Cohesin Ring

**DOI:** 10.1016/j.molcel.2020.07.013

**Published:** 2020-09-17

**Authors:** Torahiko L. Higashi, Patrik Eickhoff, Joana S. Sousa, Julia Locke, Andrea Nans, Helen R. Flynn, Ambrosius P. Snijders, George Papageorgiou, Nicola O’Reilly, Zhuo A. Chen, Francis J. O’Reilly, Juri Rappsilber, Alessandro Costa, Frank Uhlmann

**Affiliations:** 1 Chromosome Segregation Laboratory, The Francis Crick Institute, London NW1 1AT, UK; 2 Macromolecular Machines Laboratory, The Francis Crick Institute, London NW1 1AT, UK; 3 Structural Biology STP, The Francis Crick Institute, London NW1 1AT, UK; 4 Proteomics STP, The Francis Crick Institute, London NW1 1AT, UK; 5 Peptide Chemistry STP, The Francis Crick Institute, London NW1 1AT, UK; 6 Bioanalytics Unit, Institute of Biotechnology, Technische Universität Berlin, 13355 Berlin, Germany; 7 Wellcome Centre for Cell Biology, University of Edinburgh, Edinburgh EH9 3BF, UK

**Keywords:** chromosome segregation, sister chromatid cohesion, SMC complexes, ABC-ATPase, cohesin, Mis4/Scc2/NIPBL, cryo-electron microscopy, DNA-protein crosslink mass spectrometry, DNA loop extrusion, *S. pombe*

## Abstract

Despite key roles in sister chromatid cohesion and chromosome organization, the mechanism by which cohesin rings are loaded onto DNA is still unknown. Here we combine biochemical approaches and cryoelectron microscopy (cryo-EM) to visualize a cohesin loading intermediate in which DNA is locked between two gates that lead into the cohesin ring. Building on this structural framework, we design experiments to establish the order of events during cohesin loading. In an initial step, DNA traverses an N-terminal kleisin gate that is first opened upon ATP binding and then closed as the cohesin loader locks the DNA against the ATPase gate. ATP hydrolysis will lead to ATPase gate opening to complete DNA entry. Whether DNA loading is successful or results in loop extrusion might be dictated by a conserved kleisin N-terminal tail that guides the DNA through the kleisin gate. Our results establish the molecular basis for cohesin loading onto DNA.

## Highlights

• 3.9-Å cryo-EM structure of the cohesin complex in a DNA gripping state intermediate
• DNA is trapped between two gates that lead into the cohesin ring
• The kleisin N-tail guides DNA through a kleisin N-gate into the gripping state
• ATP hydrolysis opens the head gate to complete DNA entry

## Graphical Abstract

**Figure:**
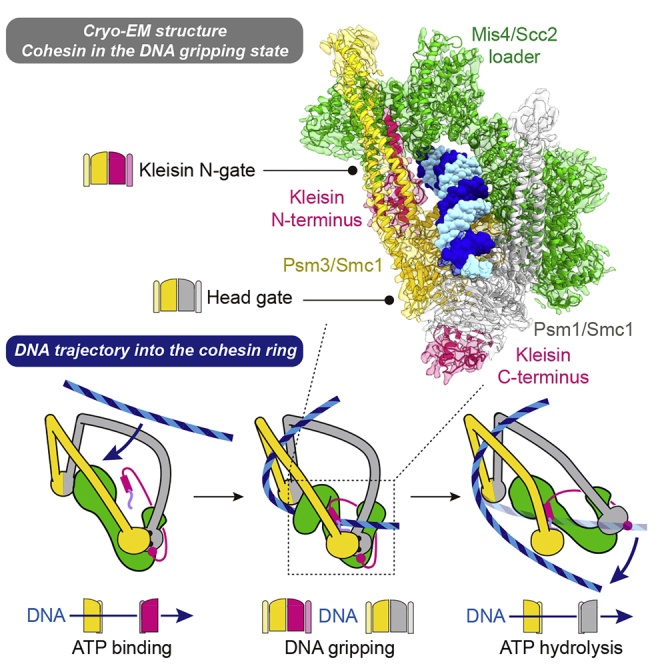

## Teaser

Cohesin is a ring-shaped protein complex that topologically entraps DNA to fulfil key functions in chromosome architecture. In a multidisciplinary approach, Higashi et al. use cryo-EM and biochemical techniques to describe how ATP-fueled structural changes of the cohesin complex drive the DNA entry reaction into the cohesin ring.

## Introduction

The structural maintenance of chromosomes (SMC) protein family is conserved from prokaryotic to eukaryotic cells, and their role in DNA organization is vital for many aspects of chromosome function (Hirano, 2016; Jeppsson et al., 2014; Uhlmann, 2016). Among the SMC complexes, cohesin establishes cohesion between replicated sister chromatids, which forms the basis for faithful chromosome segregation during cell division. Additional roles of cohesin include chromatin domain organization in interphase as well as DNA repair by homologous recombination. A characteristic feature of cohesin is its ability to bind DNA by topological embrace, which underpins sister chromatid cohesion (Haering et al., 2008). At the same time, cohesin has been seen to extrude DNA loops without a need for the ring to topologically trap DNA (Davidson et al., 2019; Kim et al., 2019). Such a loop extrusion mechanism has been proposed to underlie interphase chromatin domain organization. The molecular mechanisms by which cohesin topologically entraps DNA or extrudes a DNA loop are not yet understood.

The cohesin ring consists of three core components: two SMCs and a kleisin subunit. The two SMC subunits, Psm1^Smc1^ and Psm3^Smc3^, form long anti-parallel coiled coils that interact at one end at a dimerization motif, called the “hinge.” The SMC coiled coils show flexibility, pivoting at an “elbow” that is situated approximately halfway along their length (Anderson et al., 2002; Bürmann et al., 2019). At the other end of the coiled coil lie ATP-binding cassette (ABC)-type nucleotide binding domains, known as “heads.” These dimerize following ATP binding and disengage upon ATP hydrolysis (Hopfner et al., 2000). Α kleisin subunit, Rad21^Scc1^, bridges the two ATPase heads to complete this ring architecture. Elements close to the kleisin N terminus form a triple helix with the Psm3 “neck” where the coiled coil joins the Psm3 ATPase head. The kleisin C terminus, in turn, forms a small winged-helix domain that associates with the Psm1 head (Gligoris et al., 2014; Haering et al., 2004).

The SMC-kleisin ring is regulated by three additional HEAT repeat subunits that associate with the unstructured middle region of the kleisin. The Psc3^Scc3/STAG1/2^ subunit binds to the center of this region and is instrumental for cohesin loading and unloading (Hara et al., 2014; Li et al., 2018; Murayama and Uhlmann, 2014, 2015). Mis4^Scc2/NIPBL^ is known as the cohesin loader. It binds the kleisin upstream of Psc3^Scc3/STAG1/2^ and, together with its Ssl3^Scc4/MAU2^ binding partner, is essential for chromosomal cohesin loading. Mis4^Scc2/NIPBL^ by itself is sufficient to promote *in vitro* cohesin loading onto DNA, whereas Ssl3^Scc4/MAU2^ serves as an *in vivo* chromatin adaptor (Kikuchi et al., 2016; Muñoz et al., 2019; Murayama and Uhlmann, 2014). The HEAT subunit Pds5, in turn, competes with Mis4^Scc2/NIPBL^ for kleisin binding. Pds5 has a dual role in stabilizing loaded cohesin on DNA as well as recruiting the cohesin unloading factor Wapl (Lee et al., 2016; Ouyang et al., 2016).

Recent studies have started to shed light onto the molecular mechanism of cohesin unloading from DNA, which depends on ATP hydrolysis and, therefore, likely on dissociation of the ATPase heads (head gate opening). Pds5 and Wapl, in turn, promote dissociation of the kleisin N terminus from Psm3^Smc3^ (kleisin N-gate opening), consistent with an outward DNA trajectory through the ATPase head and kleisin N-gates (Beckouët et al., 2016; Buheitel and Stemmann, 2013; Chan et al., 2012; Murayama and Uhlmann, 2015).

How DNA enters the cohesin ring remains controversial. Cohesin loading onto DNA also depends on its ATPase and on two conserved Psm3^Smc3^ lysine residues (K105 and K106 in fission yeast) that, together with the cohesin loader, convey DNA-stimulated ATP hydrolysis (Arumugam et al., 2003; Murayama and Uhlmann, 2014, 2015; Weitzer et al., 2003). At least *in vitro*, Pds5 and Wapl also facilitate topological loading. Because these requirements are similar to those of cohesin unloading, we hypothesized that cohesin loading uses the same DNA trajectory through ATPase head and kleisin N-gates. The entry reaction would be facilitated by loader-dependent cohesin ring folding, exposing the luminal Psm3^Smc3^ K105 and K106 residues to DNA (Murayama and Uhlmann, 2015). However, *in vitro* studies suggest that ATP binding, but not hydrolysis, is required for topological cohesin binding to DNA, which is hard to reconcile with the above model (Çamdere et al., 2018; Minamino et al., 2018). Furthermore, an alternative model states that DNA enters the cohesin ring through the hinge (Gruber et al., 2006).

To understand the process of cohesin loading onto DNA, we used fluorescence resonance energy transfer (FRET) to measure conformational changes at the SMC heads. This showed that the SMC heads engage in the presence of DNA, the cohesin loader, and non-hydrolyzable ATP. We visualized this cohesin loading intermediate by cryo-electron microscopy (cryo-EM) at an average resolution of 3.9 Å. DNA is trapped between the kleisin N-gate and the head gate, whereas the cohesin loader plays a key structural role in stabilizing this state. The development of DNA-protein crosslink mass spectrometry (DPC-MS) allows us to trace the DNA trajectory, leading to a model where DNA passes through the kleisin N-gate before reaching the engaged SMC heads. DNA and the loader will trigger ATP hydrolysis and head disengagement to complete DNA entry into the cohesin ring.

## Results

### Cohesin ATPase Engagement with the Loader, DNA, and ATP

**Figure 1:**
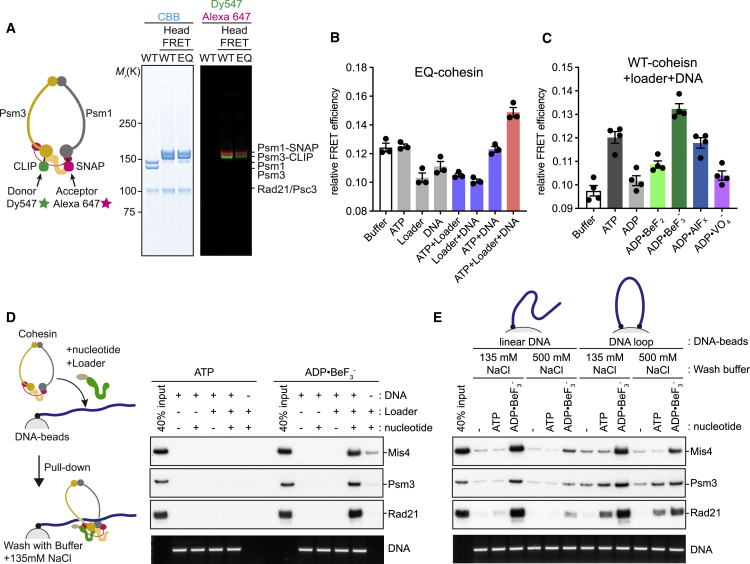
Cohesin ATPase Head Engagement Leads to a DNA Gripping State (A) Schematic of purification and labeling of wild-type (WT) and Walker B mutant (EQ) cohesin to measure FRET between the Psm1 and Psm3 ATPase heads. Purified complexes were analyzed by SDS-polyacrylamide gel electrophoresis (PAGE) followed by Coomassie blue (CBB) staining or in-gel fluorescence detection. (B) Head FRET efficiencies of EQ-cohesin with the indicated additions were calculated by dividing the Alexa 647 intensity at its emission peak by the sum of Alexa 647 and Dy547 intensities. Results from three independent repeats of the experiment and their means and standard deviations are shown. (C) Head FRET efficiencies of WT cohesin in the presence of the Mis4-Ssl3 loader, a 3-kb circular plasmid DNA and the indicated nucleotides and phosphate analogs. Results from four independent repeats of the experiment and their means and standard deviations are shown. (D) Schematic of the DNA gripping experiment. Following incubation and washes, bound protein was analyzed by SDS-PAGE and immunoblotting, and the DNA was visualized by agarose gel electrophoresis. (E) Salt sensitivity of cohesin-DNA complexes following assembly with hydrolyzable or non-hydrolyzable ATP on linear DNA and DNA loops. Following incubation and washes, the products were analyzed as in (D). See Figure S1 for further characterization of cohesin’s DNA gripping state.

To understand how ATPase head engagement by cohesin is regulated, we monitored head proximity of fission yeast cohesin using FRET. We used a tetramer complex consisting of Psm1, Psm3, Rad21, and Psc3 (Murayama and Uhlmann, 2014), including C-terminal SNAP and CLIP tags on Psm1 and Psm3, respectively, that could be specifically labeled with Dy547 and Alexa 647 fluorophores as a FRET pair (Figures 1A and S1A). The labeled cohesin displayed wild-type levels of Mis4-dependent DNA loading (Figure S1B). In the absence of ATP and DNA, cohesin exhibited measurable FRET, suggesting relative proximity between the two ATPase heads. Addition of ATP slightly increased FRET efficiency, consistent with ATP-dependent head engagement (Figure S1C). Combination of ATP with a 3-kb circular plasmid DNA and loader did not further augment the FRET signal, although addition of the Mis4-Ssl3 cohesin loader alone or in pairwise combinations with ATP or DNA reproducibly reduced FRET efficiency.

Cycles of head engagement and disengagement following ATP hydrolysis might dampen any bulk FRET changes. To prevent ATPase cycling, we purified ATP hydrolysis-deficient Walker B motif mutant cohesin (EQ-cohesin; Figure 1A). FRET changes were indeed augmented when using EQ-cohesin. Addition of Mis4-Ssl3 with or without DNA again resulted in FRET loss, suggesting that the cohesin loader has a tendency to separate the ATPase heads (Figure 1B). Strikingly, the presence of all three components—the loader, DNA, and ATP—resulted in a marked FRET increase. This suggests that head engagement is reached when all loading components come together. We observed this state only using ATP hydrolysis-deficient cohesin, indicating that head engagement is usually transient. A further implication of this observation is that, most times, the ATPase heads of wild-type cohesin are in proximity but not engaged.

To further explore the requirements for head engagement, we compared the Mis4-Ssl3 cohesin loader complex with Mis4 lacking its N-terminal Ssl3-interacting region (Mis4-N191; Chao et al., 2015). Mis4-N191 is competent in *in vitro* cohesin loading and was equally proficient in promoting head engagement (Figure S1D). On the contrary, Pds5-Wapl did not promote head engagement, revealing a mechanistic difference between loader and unloader (Figure S1E). Head engagement was equally promoted by linear or circular double-stranded DNA (dsDNA) or single-stranded DNA (ssDNA) (Figure S1F).

Head engagement could also be reached by wild-type cohesin in the presence of the loader, DNA, and non-hydrolyzable ATP analogs. A FRET increase was elicited by ADP and BeF_3_^−^, an ATP ground state mimic, or AlF_x_ (comprising a mixture of the AlF_3_ ground state and AlF_4_^−^ transition state mimics) but not the VO_4_^3−^ transition state mimic (Figure 1C). These observations suggest that SMC head engagement is reached following ATP binding in its ground state.

### Head Engagement Leads to a DNA “Gripping” State

While performing the above experiments, we noticed unusually tight cohesin binding to linear DNA in the presence of the loader and non-hydrolyzable ATP. When using linear bead-bound DNA as a substrate for cohesin loading, little cohesin is retained following incubation with the loader and ATP and a wash at physiological salt concentration (135 mM NaCl). However, when we used ADP·BeF_3_^−^ as the nucleotide, cohesin and the loader were efficiently retained on DNA (Figure 1D). Tight DNA binding was reproduced using EQ-cohesin and ATP, albeit with somewhat lower efficiency compared with wild-type cohesin and ADP·BeF_3_^−^, maybe because of residual ATP hydrolysis by EQ mutant cohesin (Figure S1G). Mis4-N191, but not Pds5-Wapl, also generated this tight DNA binding (Figure S1H), which we refer to hereafter as “DNA gripping.”

Although the gripping state tolerated physiological salt washes, it was sensitive toward higher salt concentrations (500 mM NaCl), suggesting that it arises from electrostatic interactions. Such a high-salt wash removed gripped cohesin from linear DNA but not from DNA loops with both ends tethered to the beads (Figure 1E). This suggests that, in addition to high-salt-sensitive gripping, cohesin retains a high-salt-resistant topological association with DNA, corresponding to the previously observed topological cohesin binding using non-hydrolyzable ATP (Çamdere et al., 2018; Minamino et al., 2018).

Finally, we needed to know whether the gripping state is equivalent to fully topologically loaded cohesin following ATP hydrolysis. In both cases, cohesin is retained on topologically closed DNA following high-salt washes that are usually performed on ice. However, when we incubated cohesin-DNA complexes in a high-salt buffer at 32°C for 60 min, only cohesin that was loaded by ATP hydrolysis was retained on DNA. Cohesin in the gripping state was lost following this incubation (Figure S1I). Together, these data suggest that the cohesin gripping state includes topological DNA embrace but that it is biochemically distinct from and less stable than fully topologically loaded cohesin.

### Cryo-EM Structure of Cohesin in the Gripping State

**Figure 2:**
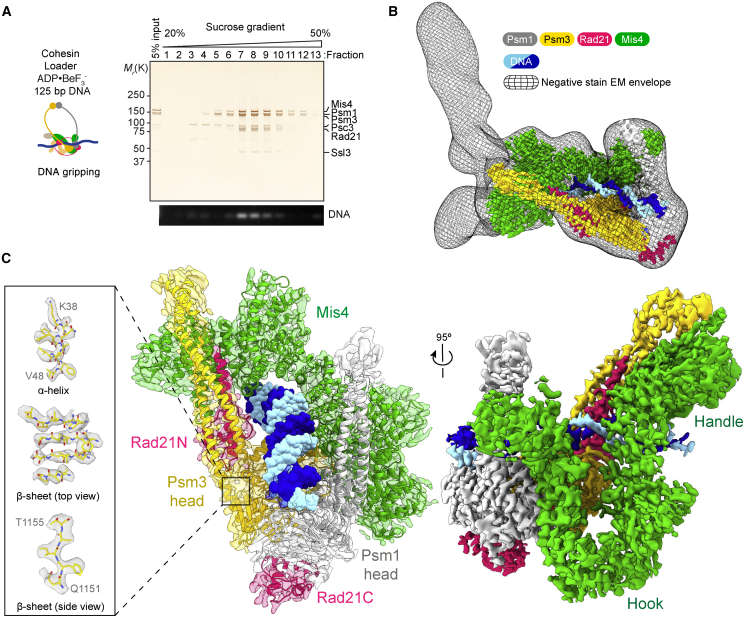
Overview Structure of Cohesin during Its Loading onto DNA (A) Schematic of EM sample preparation. The DNA gripping was separated by sucrose gradient centrifugation. The protein and DNA composition of each fraction were analyzed by SDS-PAGE followed by silver staining and agarose gel electrophoresis. Fractions 7 and 8 were used for EM analysis. (B) Superposed image of the negative staining 3D reconstruction and cryo-EM map of the cohesin core complex. (C) Two views of the 3.9-Å resolution cryo-EM map of the core complex with a transparent surface containing the atomic model (center) and a solid surface rendering (right). Three examples of secondary structure elements with resolved amino acidic side chains are shown. See also Figure S2, which documents the negative staining and cryo-EM data collection and image processing.

To understand the molecular architecture of the gripping state, we visualized this cohesin-DNA-loader complex by EM. We assembled cohesin onto a linear 125-bp dsDNA substrate in the presence of Mis4-Ssl3 and ADP·BeF_3_^–^ and separated the gripping reaction by sucrose gradient centrifugation (Figure 2A). The cohesin tetramer, loader, and DNA co-fractionated, and the peak fractions were applied to EM grids and stained with uranyl acetate. Particles were homogeneously distributed, and 2D averages revealed a Y-shaped complex (Figures S2A and S2B). A 3D reconstruction revealed two extended protrusions, bean- and rod-shaped, which asymmetrically depart from the core complex (Figures 2B and S2C–S2E). A U-shaped density was apparent as part of the core, reminiscent of the Scc2 cohesin loader subunit (Chao et al., 2017; Kikuchi et al., 2016). Other cohesin components and the DNA were harder to assign.

To visualize the cohesin-DNA complex at higher resolution, we recorded cryo-EM images of the same preparation. 2D averages revealed fine details in the core complex, whereas the protruding densities observed in the negative-stain averages were less defined. Following 3D classification and local refinement, we obtained a first 3.9-Å resolution map of this core (Figures 2C and S2F– S2L). At this resolution, secondary structure elements and large amino acid side chains became discernible, allowing us to build an atomic model, starting from docked homology models derived from available crystal structures (Gligoris et al., 2014; Haering et al., 2004; Kikuchi et al., 2016). The model covers the Psm1 head and proximal coiled coil bound to the Rad21 C terminus, the Psm3 head and neck bound by the Rad21 N-terminal domain, as well as Mis4 and 32 bp of DNA.

The DNA lies on top of the engaged ATPase heads, which are nucleotide bound and in a configuration competent for ATP hydrolysis. Mis4 clamps the DNA onto the ATPase heads, making widespread contacts with Psm1 and Psm3. This cryo-EM volume could be docked into the negative-stain map, indicating that the core of the complex overall maintains the same configuration in the room temperature-fixed sample and in the frozen-hydrated state (Figure 2B). From overlaying the two reconstructions, we see that the bean and rod in the negative-stain map contact N-terminal Mis4 and the Psm3 coiled-coil region.

### The Molecular Action of the Cohesin Loader

**Figure 3:**
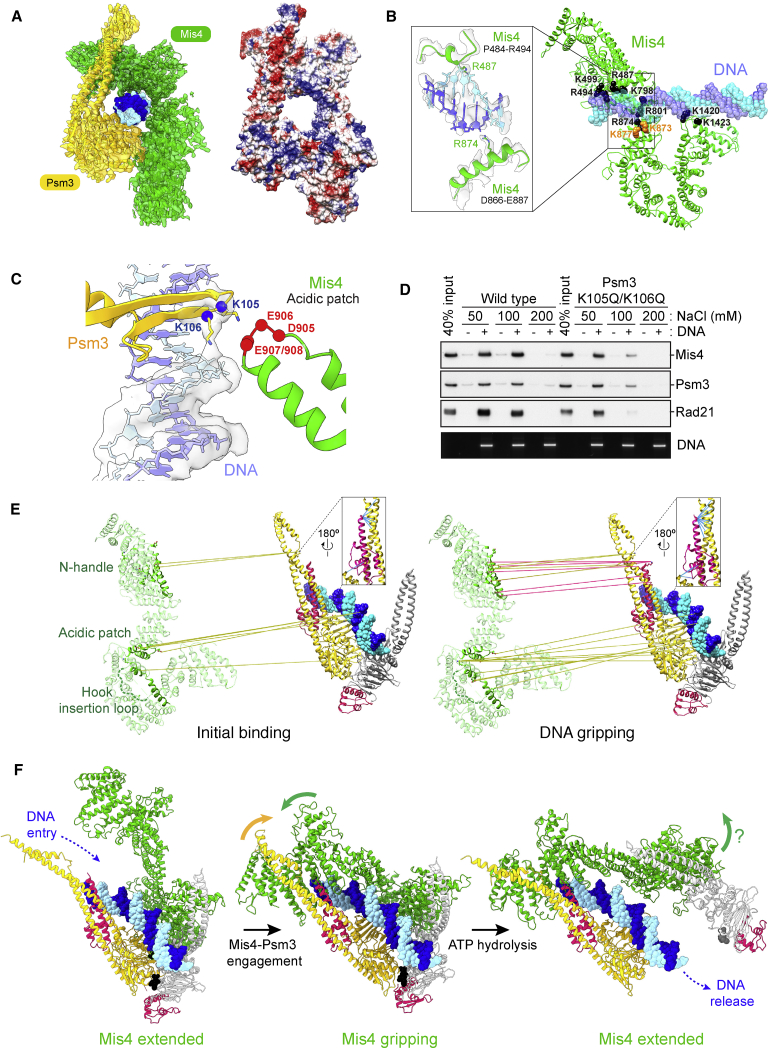
Molecular Mechanism of the Cohesin Loader (A) Psm3 and Mis4 topologically embrace DNA in the gripping state. Shown are an atomic model of Psm3, Mis4, and DNA built into the cryo-EM map (left) as well as Coulombic surface coloring for the protein component. Blue represents positively and red negatively charged amino acids. (B) Positively charged residues on the Mis4 surface, colored black, line the DNA path. The inset displays the cryo-EM map and atomic model to illustrate contacts made by R487 and R874 with the DNA. K873 and K877 are highlighted in gold. (C) Atomic model and cryo-EM map surrounding Psm3 K105 and K106 acetyl acceptor lysines, their orientation with respect to DNA, and a Mis4 acidic patch. (D) DNA gripping experiment comparing salt resistance of WT and acetyl acceptor lysine mutant (K105Q/K106Q) cohesin in the presence of ADP⋅BeF_3_^−^. (E) Comparison of CLMS contacts between initial binding and the DNA gripping state. Crosslinks between Mis4 and Psm3 (golden lines) and between Mis4 and Rad21 (red lines) were mapped onto an expanded atomic model of the DNA gripping state. Insets show crosslinks between Rad21 and the Psm3 neck (blue lines). (F) Hypothetical sequence of ATP hydrolysis-controlled Mis4 conformational changes before and after gripping state formation. The behavior of the Rad21 N terminus is explored in Figure 7. See Figure S3 for additional analyses of the DNA gripping state.

Inspection of our atomic model reveals two contact regions between Mis4 and cohesin. The U-shaped Mis4 hook binds the engaged Psm1-Psm3 ATPase heads, whereas the Mis4 N-terminal handle contacts the Psm3 neck region (Figure 2C). Mis4 and Psm3 together form a protein ring that topologically encircles the DNA (Figure 3A). The lumen of the ring is lined with positive charges mainly clustered on the Mis4 surface. A protein ring, partly formed by Mis4 in the gripping state, is unexpected and distinct from the well-established cohesin ring. Our structure explains why ATP binding and not hydrolysis is sufficient for topological DNA binding (Çamdere et al., 2018; Minamino et al., 2018). Thus, two distinct topological interactions are formed between protein and DNA during cohesin loading. One interaction is a loading intermediate, which directly involves the Mis4 loader and depends on ATP head engagement, shown here. The second interaction is the end result of the cohesin-loading reaction (Haering et al., 2008), which involves the topological entrapment in the main SMC ring and requires ATP hydrolysis.

In addition to topologically entrapping DNA, Mis4 is involved in multiple DNA contacts. Regions where the Mis4 cryo-EM density is connected with DNA include R487 and R874, where the N-terminal handle and the C-terminal hook clamp the double helix (Figure 3B). Two conserved positively charged residues, K873 and K877, map in close proximity and are required for cohesin loading onto chromosomes in *S. cerevisiae* (Chao et al., 2017). The engaged SMC heads provide an additional, composite DNA binding surface lined with conserved positively charged residues (Figure S3A). These extensive DNA contacts likely underlie the electrostatic binding of linear DNA in the gripping state.

When we superimpose the engaged DNA-bound SMC heads in our structure with the equivalent domains of Rad50 from the Rad50-Mre11-DNA complex, we find a striking overlap between the ATP-bound ATPase heads as well as the DNA (Figure S3A; Liu et al., 2016; Schüler and Sjögren, 2016). Furthermore, positively charged residues that make electrostatic contact with DNA are spatially conserved on the ATPase surface despite the limited sequence conservation between Rad50 and Psm1-Psm3. This suggests fundamental similarities in the DNA binding mechanisms of these distant SMC family relatives.

Among residues that make direct contact with the DNA is Psm3 K106, one of the two conserved acetyl acceptor lysines (Figure 3C). The second, K105, does not make obvious DNA contact. Rather, cryo-EM density inspection indicates that K105 is oriented toward a conserved acidic surface loop on Mis4 (Figures 3C and S3B). Thus, K105 and K106 emerge as a signaling node where DNA and the cohesin loader converge. This arrangement explains why ATPase stimulation of cohesin depends on the presence of DNA and the cohesin loader (Murayama and Uhlmann, 2014). To investigate the contribution of the two lysines to the gripping state formation, we mutated both residues to asparagine. Although DNA gripping was still observed with this KKQQ mutant at low salt concentration (50 mM NaCl), contact was lost at an intermediate salt concentration (100 mM), where wild-type cohesin retains tight DNA binding (Figure 3D). This observation confirms an important contribution of K105 and K106 to gripping state formation.

To further explore the Mis4-cohesin interactions, we performed protein-protein crosslinking mass spectrometry (CLMS) using a bifunctional, UV-activated (N-hydroxysuccinimide [NHS]-diazirine) crosslinker (Figure S3C). We compared the loader-cohesin contacts in the absence and presence of nucleotide, recapitulating an “initial state” before head engagement and the gripping state described in our structure. Most (97.5%) crosslinks within subunits of the cohesin core map within 25 Å when projected onto our cryo-EM atomic model, the length of the crosslinker, validating our approach (Figure S3D). Looking at intermolecular crosslinks, the Mis4 hook displayed numerous contacts with the Psm3 head in both states, although the identity of the involved amino acids changed between the two conditions (Figures 3E). We detect contacts between the Psm3 head and both flanks of the Mis4 hook, including crosslinks with a characteristic, conserved “hook insertion loop” that emerges from the C-terminal Mis4 flank and crosses the hook crevice to the N-terminal flank before looping backward (Figures 3E, S3B, and S3E). Proximity between Psm3 K105 and the Mis4 acidic patch was also confirmed in this crosslinking experiment.

In contrast to these prevalent hook interactions, CLMS contacts of the Mis4 handle with the Psm3 neck were scarce in the initial state but became prominent in the gripping state (Figure 3E). Conserved Mis4 handle residues (Figure S3B) make crosslinks with the Rad21 N-terminal domain in the gripping state that were absent in the initial state. We can rule out that lack of Mis4 handle-Rad21 interactions reflect an absence of Rad21 because Rad21-Psm3 neck crosslinks were detected in both states. These observations open the possibility that Mis4 hook engagement with the Psm3 head is created in the initial state, whereas Mis4 interactions with the Psm3 neck and Rad21 are stabilized upon ATP-dependent DNA gripping.

In our cryo-EM structure of the gripping state, Mis4 shows a striking conformational change compared with the crystal structure of free *C. thermophilum* Scc2 (Kikuchi et al., 2016). Although the U-shaped Mis4 hook can be superimposed with a root-mean-square deviation (RMSD) of only 1.0 Å, the angle at which the N-terminal handle emerges is tilted by 40° (Figure S3F). If we superpose Mis4 via the U-shaped hook domain in the X-ray form to our gripping-state structure, then the N-terminal handle becomes disengaged from the Psm3 neck and N-terminal Rad21, opening up a corridor for DNA to access the ATPase (Figure 3F, left). Given that the Mis4 N-terminal handle engages the double helix in our structure, we speculate that DNA entry itself contributes to rearranging the handle en route to gripping-state formation (Figure 3F, center). To facilitate Mis4 engagement, the Psm3 coiled coil rotates by 25° with respect to its ATPase head compared with other available structures of cohesin SMCs that were captured with engaged ATPase heads (Gligoris et al., 2014; Muir et al., 2020; Figure S3G).

Mis4 and DNA contact with Psm3 K105 and K106 in the gripping state should trigger ATP hydrolysis. To create a model for head disengagement, we first defined Psm3 with the N-terminal Mis4 handle and Psm1 with the C-terminal Mis4 hook as two separate rigid bodies. We then modeled a Mis4 reconfiguration from the gripping state back to its extended X-ray form. This pushes the ATPase heads apart, opening a path for DNA passage through the SMC head gate (Figure 3F, right). The tendency of Mis4 to increase the Psm1-Psm3 head distance in the absence of nucleotide (Figure 1B) is consistent with this scenario.

### A Hybrid Model of the Complete Cohesin Complex

To understand how loader-facilitated DNA passage through the SMC head gate contributes to topological cohesin loading, we need to place this reaction into the context of the complete cohesin complex.

**Figure 4:**
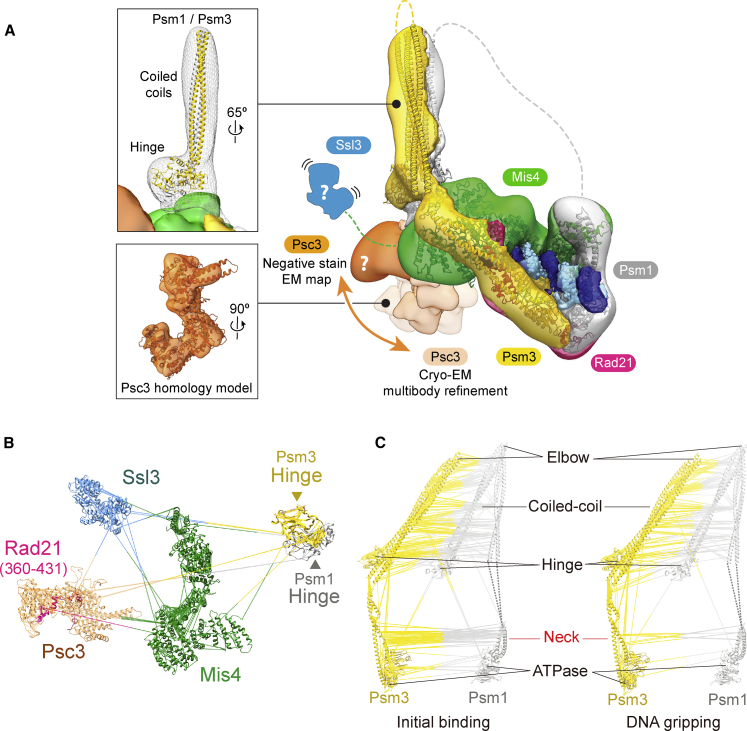
A Hybrid Structural Model of the Cohesin Complex in the Gripping State (A) Atomic model of the cohesin core docked into the negative-stain EM envelope. An atomic model of the hinge and coiled coil is placed into the rod-shaped extension. The overall density accommodates a large portion of the Psm3 coiled coil, whereas parts of Psm1 remained invisible (dashed lines). The structure of Psc3 derived from multibody refinement of the cryo-EM structure is shown, and its variable positions are indicated. The likely position of Ssl3 bound to the Mis4 N terminus is indicated. (B) Protein crosslinks between the atomic models in the gripping state, supporting the assignments in (A). (C) Comparison of protein crosslinks within and between the SMC coiled coils in the initial binding and gripping state. See Figure S4 for supporting analyses of the hybrid structural model.

By multibody refinement using masks for the cohesin core and a peripheral, Mis4-contacting density feature, we could identify a distinct rigid body. Although only resolved to ~10 Å, this feature unambiguously matches a Psc3 homology model based on the *S. cerevisiae* Scc3 crystal structure (Li et al., 2018; Figures 4A and S4A). The degree of flexibility, derived from the multibody refinement, implies a loose association with the cohesin core, at least in the state captured in our structure. In agreement with our assignment, several crosslinks in our CLMS dataset can be observed between Psc3 and Mis4 and the Psm3 head (Figures 4B and S4B). The negative-stain 3D reconstruction contains a bean-shaped feature also mapping in proximity to the Psm3-Mis4 channel (Figure 4A). Should this feature also correspond to Psc3, it would appear further tilted, suggesting a large degree of flexibility relative to the cohesin core.

The small Ssl3 subunit of the cohesin loader was part of our preparation but remained invisible in the EM structure, probably because it remains loosely tethered to the core complex. Nevertheless, our CLMS analysis revealed numerous crosslinks involving this subunit (Figures 4B and S4B). As expected, Ssl3 efficiently crosslinks with the Mis4 N terminus it encapsulates (Chao et al., 2015; Hinshaw et al., 2015). Further Ssl3 crosslinks were detected with Mis4, Psc3, and Psm3, consistent with a flexible position of Ssl3 on the posterior face of the cohesin core. Given the low resolution, we cannot exclude that the bean-shaped feature corresponds to Ssl3, not Psc3, in our negative-stain reconstruction. The implications of this positioning for interactions with chromatin receptors and the *in vivo* mechanism of cohesin loading remain to be explored in the future.

A feature unique to the negative-stain reconstruction is a prominent rod that projects from between the Mis4 handle and the bean-shaped feature. Its dimensions are well compatible with a model of the cohesin hinge connected to the SMC coiled coils. Atomic docking indicates that the Psm3 hinge makes direct contact with Mis4. Crosslinks detected between the hinge and Mis4 and Psc3 support this assignment (Figures 4A, 4B, and S4B).

The hinge is connected with the ATPase heads via long stretches of coiled coil. Numerous intramolecular crosslinks in our CLMS dataset reflect coiled-coil formation (Figures 4C and S4C). Intermolecular crosslinks between Psm1 and Psm3 suggest that both arms extend in parallel from the hinge and, in the gripping state, interact with each other up to about two-thirds of their length. However, numerous intra- and intermolecular crosslinks cannot be simply explained by an extended coiled-coil configuration because the distance between crosslinked residues largely exceeds the linker length. The distance constraints can be fulfilled if the coiled coil turns back on itself with an inflection point at its predicted elbow (Bürmann et al., 2019; Figures 4C, S4C, and S4D). Consistent with folding at the elbow, we also observed crosslinks where the hinge is expected to touch down on the coiled coil. These crosslinks all emanate from the same hinge face, suggesting that folding is directional. Based on these constraints, we can model a folded Psm3 coiled coil, showing good agreement with the negative-stain envelope (Figure 4A). We did not observe continuous EM density for the Psm1 coiled coil, whose path, therefore, remains tentative. A single coiled-coil feature is likely too thin to be visualized by negative-stain EM. In addition, SMC coiled coils are flexible and adopt a wide range of conformations (Anderson et al., 2002; Eeftens et al., 2016). Hence, our structural model reflects the observed positioning but does not suggest a fixed orientation for the coiled coil in the gripping state.

When nucleotide was omitted in the initial state, coiled-coil and hinge crosslinks were also indicative of elbow inflection. Intermolecular Psm1-Psm3 crosslinks in the initial state extended farther along the coiled coil and toward the heads (Figure 4C). This observation could indicate a defined state where the Psm1-Psm3 necks come in closer proximity. Alternatively, these Psm1-Psm3 crosslinks could report on an enhanced structural flexibility in the absence of nucleotide. In support of the latter scenario, nucleotide-dependent head engagement in the gripping state forces the Psm1 and Psm3 necks in a divergent configuration, precluding coiled-coil interactions proximal to the heads.

### The Kleisin Path in the Gripping State

**Figure 5:**
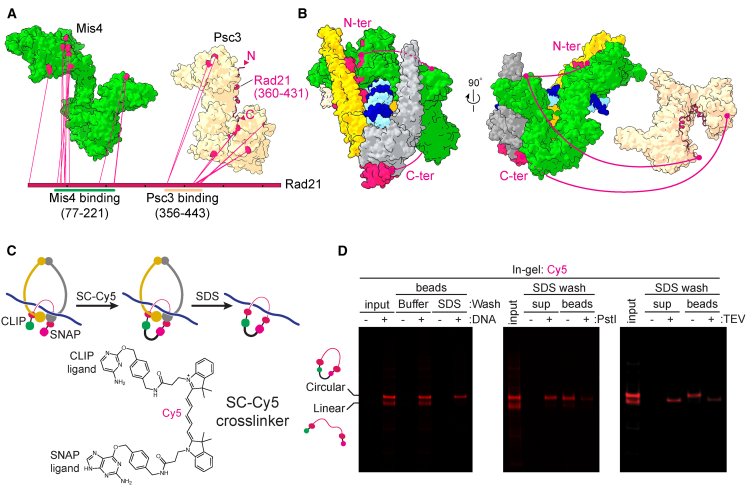
The Kleisin Path in the Gripping State (A) Crosslinks of Rad21 with Mis4 and Psc3 in the gripping state mapped onto their atomic models. Rad21 amino acids 360–431 are modeled based on the crystal structure of human SA2 bound to Rad21 (PDB: 4PK7). (B) Crosslinks from (A) mapped onto the structures of gripping state complex components, suggesting a likely kleisin path (red line). (C) Schematic of the kleisin circularization experiment. A gripping reaction was performed with Rad21 carrying N- and C-terminal CLIP and SNAP tags using a DNA loop substrate on beads. The CLIP and SNAP tags were covalently crosslinked by SC-Cy5. (D) In-gel Cy5 detection of the experiment in (C). SC-Cy5 was added to the input proteins or following gripping state assembly on the DNA beads. Beads were then washed with buffer or SDS (left). After the SDS wash, DNA beads were treated with PstI restriction endonuclease (center) or TEV protease (right), and bead-bound and supernatant fractions were analyzed. See Figure S5 for supporting information regarding the kleisin path.

A crucial component of cohesin is its kleisin subunit Rad21, which bridges the ATPase heads. Although we can see the kleisin N terminus engaged with the Psm3 neck as well as the C-terminal winged helix domain bound to the Psm1 head (Figure 2C), the kleisin middle region is not resolved in our structure, as expected from the paucity of predicted secondary structure elements. To trace the kleisin path, we again turned to our CLMS dataset, which contained sequential crosslinks between Rad21 and the two HEAT subunits Mis4 and Psc3 (Figure 5A). As expected (Hara et al., 2014; Kikuchi et al., 2016; Li et al., 2018), Rad21 amino acids 77–221 line the Mis4 handle before crossing over to the C-terminal flank of the Mis4 hook. The central amino acids 356–443, in turn, follow the Psc3 body in an N- to C-terminal direction. When we project this kleisin path onto our hybrid model of the cohesin complex, this trajectory suggests that the kleisin has encircled the DNA in cohesin’s gripping state (Figure 5B).

To probe this suggested kleisin topology with respect to the DNA, we designed an experiment to covalently join the kleisin N and C termini, fused to CLIP and SNAP tags, respectively, in the gripping state. This should result in topological DNA entrapment by the kleisin (Figure 5C). To covalently link the two tags, we chemically synthesized a crosslinker in which a SNAP substrate is linked to a CLIP substrate via a Cy5 dye moiety (SC-Cy5; Figures 5C and S5A). The combined length of these components is sufficient to bridge the kleisin N and C termini along the shortest distance to enclose the DNA but insufficient to entrap the DNA using alternative topologies (Figure S5B).

We then carried out a DNA gripping reaction using a DNA loop substrate attached to magnetic beads. Following the gripping reaction, we added SC-Cy5. This resulted in approximately equal proportions of linear Rad21, labeled at one or both ends, and circularized Rad21. The latter was identified by its retarded gel mobility. Following denaturation in buffer containing SDS, circularized Rad21, but not linear Rad21 or Psm3, remained bound to the DNA beads (Figures 5D and S5C). The topological nature of circularized Rad21 binding to the DNA was confirmed by cleaving the DNA using a restriction endonuclease or Rad21 cleavage at an engineered tobacco etch virus (TEV) protease recognition site, both of which resulted in Rad21 elution from the beads (Figure 5D). This result confirms that the kleisin indeed encircles the DNA in the gripping state. We used a similar approach with two pairs of SNAP and CLIP tags to covalently close the SMC heads and hinge simultaneously, which revealed that DNA is not yet entrapped within the circularized SMC ring (Figures S5D–S5F).

### The DNA Trajectory into the Cohesin Ring

**Figure 6:**
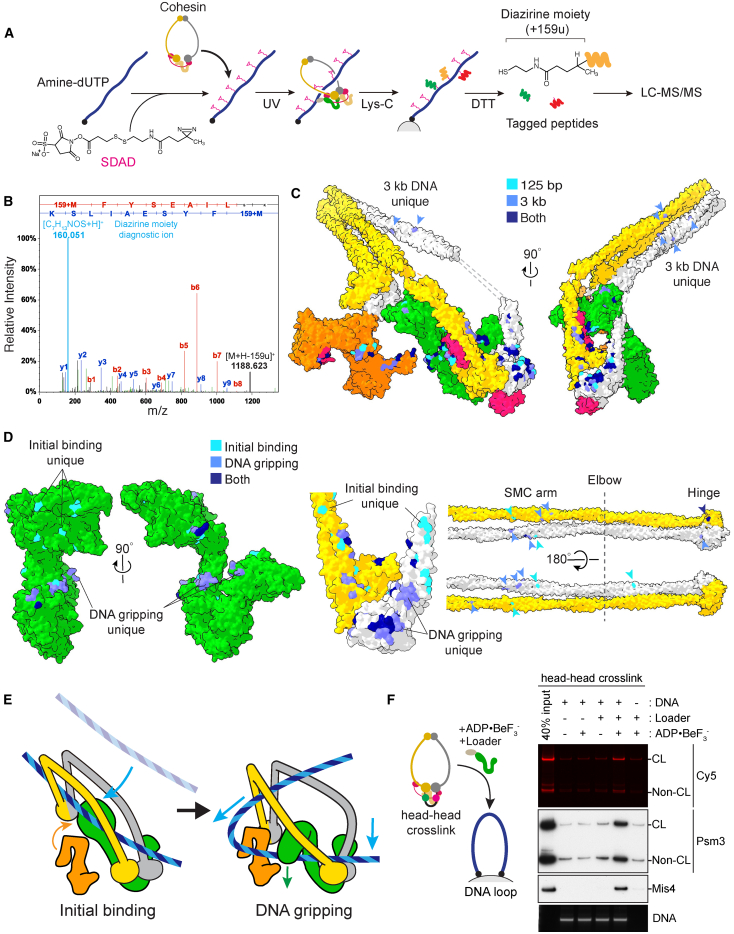
The DNA Trajectory into the Cohesin Ring (A) Schematic of the DPC-MS workflow. See the main text for details. (B) A representative mass spectrum of a peptide containing a diazirine mass tag. The diagnostic 159u ion is highlighted. (C) DNA crosslinks of a 125-bp linear DNA in the gripping state, shown on the surface of the hybrid model (light blue), compared with crosslinks observed with a 3-kb circular plasmid DNA (medium blue). Crosslinks in common are shown in dark blue. (D) DNA crosslinks in the initial DNA binding state (light blue) are compared with those in the gripping state (medium blue); those in common are shown in dark blue. Arrowheads highlight crosslinks along the SMC coiled coils and hinge. (E) A model of the DNA trajectory from initial binding toward the gripping state, based on the observed DNA contacts. (F) DNA gripping experiment using head-head crosslinked cohesin. Psm1-SNAP Psm3-CLIP cohesin was treated with SC-Cy5 to close the head gate before the gripping reaction. DNA-bound proteins were analyzed by immunoblotting and in-gel Cy5 detection. See Figure S6 for additional DPC-MS results.

Taking account of the kleisin path established above, our cryo-EM structure describes the double helix entrapped between the kleisin and ATPase head gate. However, the structure does not discriminate between two possible DNA access routes. Does the DNA enter from the bottom of the ATPase, having passed through the head gate, or, rather, from the top of the ATPase and through the kleisin N-gate? By inspecting our structure alone, we can also not rule out that a short duplex DNA segment occupying the Mis4-Psm3 cavity is an *in vitro* artifact. To explore how DNA reaches the gripping state, we developed a protocol for DPC-MS (Figure 6A).

To mark the DNA binding site on a protein, we designed a photo-crosslinkable DNA probe. First, amine-deoxyuridine triphosphate (amine-dUTP) is incorporated in place of deoxythymidine triphosphate during DNA synthesis. The amino groups are then decorated with a bifunctional succinimidyl-SS-diazirine (SDAD) crosslinker. Nucleoprotein complexes are assembled with this probe and UV-irradiated to induce DPC. Proteins are then digested using the Lys-C endopeptidase, and the DNA covalently linked to peptide fragments is isolated by biotin affinity pull-down. Finally, the disulfide bond within the SDAD crosslinker is cleaved under reducing conditions to elute the recovered peptides. These proteolytic fragments retain a characteristic mass tag of +159u at the crosslink position that can be identified by liquid chromatography-tandem MS (LC-MS/MS), mapping next to a diagnostic 159u peak that stems from loss of the mass tag upon peptide fragmentation (Figure 6B).

We first performed DPC-MS analysis using a derivatized 125-bp linear DNA probe based on our cryo-EM structure. The observed DPCs identified surface-exposed amino acids that map in proximity to the double helix in our structure along Mis4, Psm3, Psm1, as well as Psc3 (Figures 6C and S6A).

Next we repeated the DPC-MS analysis of the gripping state with a 3-kb covalently closed circular dsDNA as the substrate. Comparison with the short linear DNA revealed a near-identical range of DPCs (Figures 6C and S6A). We conclude that the DNA position observed in our cryo-EM structure is a fair reflection of that reached by a topologically closed DNA that reflects a more natural substrate. Additional DNA contacts were observed with long circular DNA involving the Psm1 and Psm3 coiled coil. These contacts inform us regarding a likely path a longer DNA takes in the gripping state (see below).

To understand the trajectory taken by DNA to reach the gripping state, we compared DPCs in the initial binding state where ATP is omitted and the nucleotide-bound gripping state. To ensure that DNA remains in a true initial state and does not reach an undesired post-hydrolysis state, we used the long circular DNA substrate in this assay. Unique DNA crosslinks were identified in the no-nucleotide state that map above the ATPase head, whereas the ATPase heads are a major DNA interaction site in the gripping state (Figures 6D and S6B). On Mis4, initial state crosslinks map on top of the handle, whereas the gripping state crosslinks line the hook. These observations suggest that DNA reaches the gripping state by approaching Mis4 and the ATPase heads from the top.

We also recorded several DNA contacts along the SMC coiled coils and hinge in the initial and the gripping state. All hinge crosslinks map on a solvent-exposed hinge surface opposite to that engaged in protein-protein contacts with the cohesin core (Figures 6D). These interactions further support a likely DNA entry path from the top of the ATPase (depicted schematically in Figure 6E).

Our proposed model for DNA entry makes the prediction that DNA would not traverse the head gate on its way to the gripping state. To test this prediction, we prepared cohesin with SNAP and CLIP tags fused to the Psm3 and Psm1 C termini, respectively. SC-Cy5 addition yielded covalent head gate closure in approximately half of the cohesin population (Figure S6C). This mixture was employed in a DNA gripping reaction using a DNA loop substrate on magnetic beads. The efficiency of DNA engagement remained unchanged irrespective of whether the SMC head gate was open or closed (Figure 6F). This observation suggests that the DNA does not need to traverse the SMC head gate to reach the DNA gripping state, further supporting DNA access from the top of the ATPase.

### ATP-Dependent Kleisin N-gate Opening

**Figure 7:**
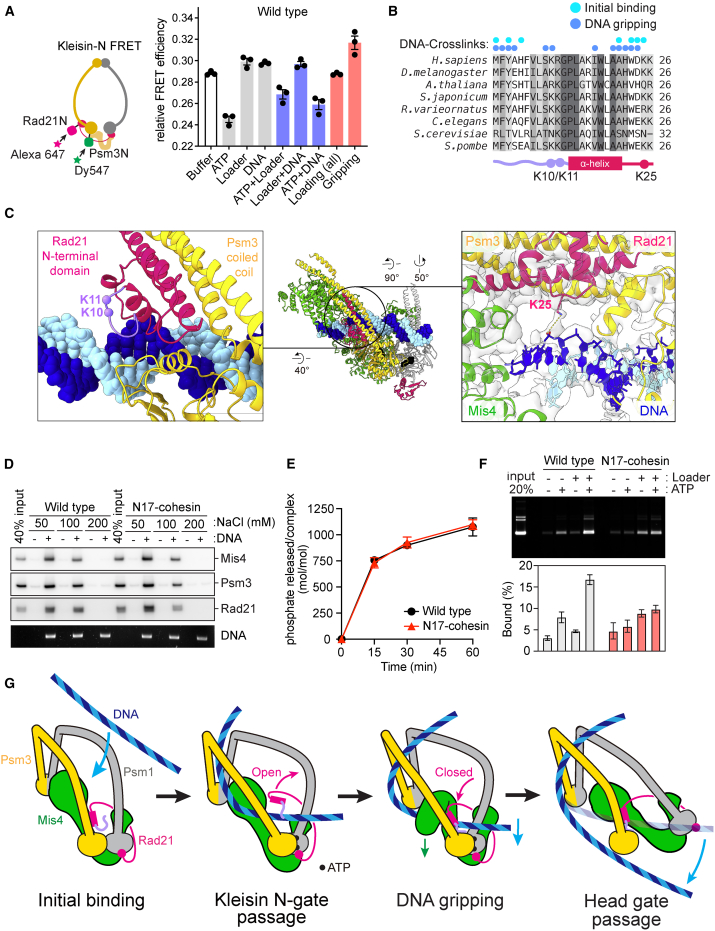
A Kleisin N-terminal Tail Guides DNA into the Cohesin Ring (A) Schematic of the kleisin N-gate FRET construct. FRET efficiencies at the kleisin N-gate were recorded under the indicated conditions using a 3-kb plasmid DNA as a substrate. ADP⋅BeF_3_^−^ was used in the gripping incubation. Results from three independent repeats and their means and standard deviations are shown. (B) Sequence alignment of the cohesin N-tail. Positions of DNA crosslinking in the initial binding and the DNA gripping state are indicated. (C) Atomic model of the Rad21 N-tail (left), showing the conserved K10 and K11 residues relative to the DNA. A magnified view around K25 is also shown (right), including the cryo-EM density. (D) Comparison of WT and N17-cohesin in a DNA gripping experiment. Following reaction with a bead-bound DNA loop substrate and washes, the bead-associated proteins and DNA were analyzed by immunoblotting and gel electrophoresis. (E) Comparison of ATP hydrolysis by WT and N17-cohesin in the presence of the loader and a 3-kb plasmid DNA. Shown are the means and standard deviations from three independent experiments. (F) Loading of WT and N17-cohesin onto a 3-kb plasmid DNA. Following the loading reaction, cohesin was immunoprecipitated and washed with buffer containing 750 mM NaCl, and recovered DNA was analyzed by agarose gel electrophoresis. Shown are the means and standard deviations from three independent experiments. (G) A model for DNA entry into the cohesin ring. The kleisin N-tail guides DNA through the kleisin N-gate before DNA reaches the ATPase heads. ATP hydrolysis and passage through the head gate completes DNA entry. See Figure S7 for further analyses of the kleisin N-tail and Video S1 for an animated model of DNA entry into the cohesin ring.

If DNA accesses the ATPase from the top, then the kleisin N-gate must open to let the DNA enter before the kleisin can encircle DNA in the gripping state. To understand how the kleisin N-gate is regulated, we performed FRET measurements between donor and acceptor fluorophore-labeled CLIP and SNAP tags attached to the Psm3 and Rad21 N-termini (Figures 7A and S7A). A cohesin tetramer in the incubation buffer displayed measurable FRET, consistent with proximity. Addition of ATP with or without DNA and the loader led to FRET loss, suggestive of kleisin N-gate opening. In contrast, addition of DNA or the loader in the absence of ATP did not induce a FRET change. Only during gripping state formation in the presence of the loader, DNA, and non-hydrolyzable ATP did we observe a distinct FRET increase, consistent with kleisin N-gate closure in this state. These observations suggest that ATP binding promotes kleisin N-gate opening and confirm that the gate is closed again in the gripping state.

To evaluate how ATP opens the kleisin N-gate, we asked whether ATPase head engagement or ATP hydrolysis is required in this process. To this end, we repeated the FRET experiment using signature motif mutant cohesin, defective in head engagement (Hopfner et al., 2000). This mutant maintained FRET even following ATP addition (Figure S7B). On the other hand, EQ-cohesin, defective in ATP hydrolysis, displayed reduced FRET and recapitulated ATP-dependent kleisin N-gate opening. These observations suggest that ATPase head engagement triggers kleisin N-gate opening but that the gate closes again when the loader clamps DNA upon gripping state formation.

### A Kleisin N-tail Guides DNA into the Cohesin Ring

The evidence acquired so far indicates that DNA must diffuse through the kleisin N-gate before reaching the gripping state. However, merely based on our structure, it is unclear what prevents DNA from reaching the gripping state without traversing the kleisin N-gate (Figure S7C). Our attention was drawn to the extreme N-terminal 12 amino acids of Rad21 that precede the α helix that forms the kleisin N-gate. This N-tail was crosslinked with DNA in our DPC-MS experiments in the initial as well as in the gripping state (Figure 7B). The N-tail is conserved among kleisins throughout evolution, including a series of positively charged residues (Figure S7D). The cryo-EM map was of sufficient quality to build amino acids 5–12 of the Rad21 N-tail, forming a loop wedged between the Psm3 ATPase head and the DNA. Two conserved lysines, 10 and 11, point toward the DNA. Although their distance is too far to maintain direct DNA contact in the gripping state, lysine 10 is among the residues crosslinked with the DNA (Figure 7C, left). Another conserved positive residue following the triple helix, lysine 25, directly engages DNA in our structure (Figure 7C, right). The N-tail is held in place by an extended loop projecting from the Psm3 ATPase head that is specific to Psm3 and much shorter in Psm1 (Figure S7E).

If the kleisin N-tail binds DNA when the kleisin N-gate opens and maintains DNA contact at least until the Rad21-Psm3 triple helix structure starts to reform, this will have guided the DNA through the kleisin gate on its way to the gripping state. To analyze the contribution of the kleisin N-tail to cohesin function, we purified a cohesin complex lacking Rad21 amino acids 1–17 (N17-cohesin; Figure S7F). When we included N17-cohesin in a DNA gripping experiment, this complex bound DNA similarly tightly compared with wild-type cohesin (Figure 7D). Likewise, the levels of DNA and loader-stimulated ATP hydrolysis were equal when using wild-type or N17-cohesin (Figure 7E). This suggests that the kleisin N-tail affects neither the tight DNA binding associated with gripping-state formation nor following ATP hydrolysis.

We then investigated the contribution of the kleisin N-tail to topological cohesin loading onto DNA. Following incubation in the presence of loader and ATP, we recovered cohesin by immunoprecipitation and assessed topological, high-salt-resistant DNA binding. N17-cohesin showed a substantially reduced ability to retain DNA compared with wild-type cohesin, indicating that topological cohesin loading onto DNA was unsuccessful (Figure 7F). From these results, we conclude that the Rad21 N-tail has a crucial role in guiding DNA to successful DNA entry into the cohesin ring (Figure 7G; Video S1).

## Discussion

We used a combination of biochemical and structural approaches to learn how DNA enters into the cohesin ring. FRET measurements at the cohesin ATPase heads led us to discover a DNA gripping intermediate that forms when cohesin comes together with the loader, DNA, and non-hydrolyzable ATP. The gripping complex proved to be a suitable target for cryo-EM imaging and allowed us to generate an atomic model of key elements of this cohesin loading intermediate. To understand where this intermediate lies during the cohesin loading reaction onto DNA, we utilized additional biochemical tools, including DPC-MS. These approaches allowed us to trace the DNA trajectory. We also identified previously uncharacterized functional elements of cohesin that are important for successful topological entry. Among these, the kleisin N-tail opens up a dichotomy of the DNA entry reaction that might be important for understanding cohesin’s alternative role in loop extrusion.

### The DNA Trajectory into the Cohesin Ring

In the gripping state, we find the DNA trapped between two gates that lead into the cohesin ring: the kleisin N-gate and the ATPase head gate. Given this topology, the DNA must have passed one of the two gates but not yet the other. Numerous lines of evidence indicate that the DNA arrived from the top of the ATPase and that it passed the kleisin N-gate. Subsequent passage through the head gate is then required to complete topological entry. Although we have not yet directly observed this step, the ATPase heads in the gripping state are ATP bound and appear to be competent for ATP hydrolysis, which is expected to trigger head gate opening.

Using a FRET-based assay, we established that kleisin N-gate opening is the consequence of ATP-dependent SMC head engagement, consistent with recent structural observations of engaged cohesin and condensin ATPase heads with an open N-gate (Hassler et al., 2019; Muir et al., 2020). Our cryo-EM structure indicates that, by the time DNA reaches the gripping state, the kleisin N-gate is closed again. In this configuration, the DNA itself contributes to keeping the gate shut by directly contacting the kleisin N-terminal domain and locking it into the triple helix with the Psm3^Smc3^ neck. ATP hydrolysis can then open the ATPase head gate, whereas the kleisin N-gate remains shut. This structural model provides an explanation for two interlocking gates through which DNA enters the cohesin ring, only one of which can be open at any one time. We hypothesized previously that DNA enters the cohesin ring through interlocking kleisin N- and head gates (Murayama and Uhlmann, 2015). Our molecular knowledge now allows us to establish the correct order of events that lead to DNA entry (Figure 7G; Video S1). The ability of ssDNA to promote head engagement opens the possibility that ssDNA-directed second DNA capture follows a similar trajectory (Murayama et al., 2018).

An alternative model for DNA loading states that DNA passes a hinge gate (Buheitel and Stemmann, 2013; Gruber et al., 2006). This model is based on two observations. First, DNA entry into the cohesin ring is blocked by ligand-induced dimerization of ectopic hinge insertions, which has been interpreted to demonstrate DNA passage through the hinge. Our structure opens up an alternative explanation. The hinge makes extensive contact with Mis4^Scc2/NIPBL^, Psc3^Scc3/STAG1/2^, as well as the coiled-coil arms. If the dimerized hinge insertions interfered with one or more of these interactions, then this could compromise coiled-coil folding, which, in turn, is crucial for DNA entry via the kleisin N-gate. A second result is, at first sight, incompatible with DNA entry through the kleisin N-gate. An Smc3-kleisin fusion protein, in which the two subunits cannot separate, remains able to load onto chromosomes (Buheitel and Stemmann, 2013; Gruber et al., 2006). However, the Smc3-kleisin fusion in itself will not block operation of the kleisin N-gate or subsequent DNA passage through the head gate. Indeed, a functional kleisin N-gate remains required for the viability of Smc3-kleisin fusion strains (Guacci et al., 2019). We therefore suggest that the fusion does not impede the loading reaction but, rather, results in a loading product in which the linker sequence between Smc3 and the kleisin adds an additional DNA embrace. Future experiments are required to clarify the topology of the DNA loading products obtained with an Smc3-kleisin fusion protein.

### The Role of the Cohesin Loader and of Other HEAT Repeat Subunits

Our structure shows how the Mis4^Scc2/NIPBL^ cohesin loader aids the topological loading reaction in several ways. Compared with its previously reported extended conformation, the loader in the gripping state has undergone a striking conformational change, contributing to the topological enclosure that holds DNA locked against the ATPase heads. Together with the DNA, the loader also engages the Psm3^Smc3^ acetyl acceptor lysines to trigger ATP hydrolysis and SMC head gate opening. Following ATP hydrolysis, we propose that the loader returns to its extended form, promoting head separation and DNA passage through the head gate. Despite these multiple ways in which the loader facilitates DNA entry, cohesin retains basal topological loading potential without the loader (Murayama and Uhlmann, 2014). Indeed, our FRET results indicate that Kleisin N-gate opening by head engagement is independent of the loader. In this scenario, arrival of the DNA alone might be sufficient to close the N-gate before ATP hydrolysis completes DNA entry at a reduced rate.

In addition to the Mis4^Scc2/NIPBL^ loader, the Psc3^Scc3/STAG1/2^ subunit is instrumental for cohesin loading onto DNA *in vivo* and *in vitro* (Murayama and Uhlmann, 2014; Tóth et al., 1999). Our structure shows that Psc3^Scc3/STAG1/2^ is positioned behind the loader in the gripping state. Furthermore, we observe DNA crosslinks consistent with a crystallographically observed DNA interaction (Li et al., 2018) in the initial DNA binding as well as the gripping state. Based on this positioning, we propose that Psc3^Scc3/STAG1/2^ plays a role in attracting and positioning the DNA as it approaches between the coiled-coil arms and moves toward the ATPase (Figure 6E). The molecular mechanism by which Psc3^Scc3/STAG1/2^ contributes to cohesin function remains to be explored further.

Cohesin’s third HEAT repeat subunit, Pds5, is thought to replace Mis4^Scc2/NIPBL^ following loading (Murayama and Uhlmann, 2015; Petela et al., 2018). Pds5 has a similar overall shape as Mis4 (Lee et al., 2016; Ouyang et al., 2016). The conserved Psm3-contacting residues in Mis4’s handle are also found in Pds5 (Figure S3B), suggesting that aspects of its cohesin engagement are likely conserved. However, unlike the loader, Pds5 does not stimulate ATP hydrolysis by cohesin (Murayama and Uhlmann, 2015), and our FRET results indicate that Pds5 fails to promote ATPase head engagement in the presence of DNA and non-hydrolyzable ATP. Instead, we speculate that Pds5 might block head engagement, preventing spontaneous kleisin N-gate opening. If kleisin N-gate opening through head engagement is no longer possible in the presence of Pds5, then an alternative way to operate the gate becomes necessary. This could be the role of Wapl that is recruited by Pds5. This scenario provides a rationale for how Pds5 could stabilize cohesin on chromosomes as well as render it competent for Wapl-regulated unloading, which could follow a similar trajectory through sequential kleisin N- and head gates. How Pds5 differs from the Mis4^Scc2/NIPBL^ loader and how their alternating association with the cohesin complex is controlled remains to be investigated.

### The Kleisin N-tail and Its Implication for Successful DNA Entry

While performing DNA-protein crosslink experiments, we noticed crosslinks with a conserved kleisin N-tail that has previously received little attention. This tail lies in proximity of the Psm3 head when the kleisin N-gate closes. If the N-tail maintains DNA contact during the transition from an open to a shut kleisin N-gate, then the tail will have guided the DNA through the kleisin gate. In support of this notion, we find that the N-tail is key to successful DNA entry into the cohesin ring. A sister chromatid cohesion defect observed in *Drosophila melanogaster* cells carrying an N-tail mutation is consistent with such a role (Ribeiro et al., 2016).

Why is a kleisin N-tail required to guide DNA through the kleisin N-gate? The structured components of the DNA gripping state have no obvious mechanism for sensing whether DNA has passed the kleisin N-gate (Figure S7C). Only our kleisin circularization experiment revealed that the DNA has, in fact, traversed the gate. We therefore speculate that, under certain conditions, DNA might reach the gripping state without having passed the kleisin N-gate. In this case, acetyl acceptor lysine engagement by DNA and the loader still triggers ATP hydrolysis, but the outcome of head disengagement will be diametrically different; without having passed the kleisin N-gate first, DNA cannot enter the cohesin ring. Following ATP hydrolysis, the loader would revert to its extended configuration, which might alter hinge interactions with the cohesin core and favor a transition from bent to straight SMC coiled coils. Provided that Psc3 retains association with the hinge and DNA during this process, cohesin would nucleate a DNA loop (Figure S7C). Were such abortive DNA entry reactions to repeat, this could lead to expansion and extrusion of the loop. DNA would not have passed any cohesin gate, consistent with experimental observations of loop extrusion by cohesin (Davidson et al., 2019). Whether DNA can reach the gripping state without passing the N-gate under physiological conditions remains an important question to explore.

While our study was under review, a structure of the human cohesin complex with its NIPBL loader was reported in a similar DNA gripping state (Shi et al., 2020). The human gripping state structure includes a conformational change of the loader relative to its previously observed crystal structure form, similar to what we observed for the fission yeast cohesin complex. While it went unnoticed by the authors, this observation opens the possibility that the molecular mechanism of DNA entry into the cohesin ring is conserved between fission yeast and human. Real-time structural and biophysical observations of cohesin during DNA entry will further elucidate this crucial process for genome stability.

## STAR★Methods

### Key Resources Table

**Table:** 

REAGENT or RESOURCE	SOURCE	IDENTIFIER
**Antibodies**		

Mouse monoclonal anti-V5	Bio-Rad	Cat# MCA1360
Mouse monoclonal anti-HA (12CA5)	Sigma-Aldrich	Cat# 11583816001
Mouse monoclonal anti-E2a (5E11)	Abcam	Cat# ab977
Rabbit polyclonal anti-Rad21 (fission yeast)	BioAcademia	Cat# 63-139
Anti-rabbit IgG (HRP-conjugated)	GE Healthcare	Cat# NA934-1ML
Anti-mouse IgG (HRP-conjugated)	GE Healthcare	Cat# NA931

**Chemicals, Peptides, and Recombinant Proteins**		

Phenylmethylsulfonyl fluoride (PMSF)	Sigma-Aldrich	Cat# 11359061001
cOmplete EDTA-Free Protease Inhibitor Cocktail	Sigma-Aldrich	Cat# 11873580001
CLIP-Surface 547	New England BioLabs	Cat# S9233S
SNAP-Surface Alexa Fluor 647	New England BioLabs	Cat# S9136S
BC-NH2	New England BioLabs	Cat# S9236S
BG-NH2	New England BioLabs	Cat# S9148S
DTT	Sigma-Aldrich	Cat# 43815-5G
BSA	ThermoFisher	Cat# AM2616
poly-dIdC:dIdC	Sigma-Aldrich	Cat# P4929-10UN
biotin	Sigma-Aldrich	Cat# B4501-1G
ATP	Sigma-Aldrich	Cat# A2383
ADP	Sigma-Aldrich	Cat# A2754
Beryllium sulrate tetrahydrate	VWR international LTD	Cat# 16104.14
Sodium fluoride 0.5 M Solution	Sigma-Aldrich	Cat# 67414-1ML-F
Aluminum chloride	Sigma-Aldrich	Cat# 449598-5G
Sodium Orthovanadate	New England BioLabs	Cat# P0758S
InstantBlue	Sigma-Aldrich	Cat# ISB1L-1L
SYBR Gold Nucleic Acid Gel Stain	ThermoFisher	Cat# S11494
Protease K	TaKaRa	Cat# 9034
AcTEV protease	ThermoFisher	Cat# 12575015
PstI-HF	New England BioLabs	Cat# R3140S
T7 DNA polymerase	New England BioLabs	Cat# M0274S
CloneAmp HiFi PCR Premix	TaKaRa	Cat# 639298
GoTaq Taq G2 DNA Polymerase	Promega	Cat# M7845
Deoxynucleotide Set	Sigma-Aldrich	Cat# DNTP100-1KT
Aminoallyl-dUTP	Stratech Scientific Ltd	Cat# NU-803S-JEN-10ul
SDAD (NHS-SS-Diazirine)	ThermoFisher	Cat# 26169
SDA (NHS-Diazirine)	ThermoFisher	Cat# 26167
Lysyl EndopeptidaseR (Lys-C)	FUJIFILM	Cat# 129-02541
Ammonium bicarbonate	Sigma-Aldrich	Cat# 09830-500G
Fission yeast cohesin (Psm1-Psm3-Rad21-Psc3)	Murayama and Uhlmann, 2014	N/A
Fission yeast EQ-cohesin (Psm1E1161Q-Psm3E1128Q- Rad21-Psc3)	Murayama and Uhlmann, 2015	N/A
Fission yeast KKQQ cohesin (Psm1-Psm3K105Q/K106Q- Rad21-Psc3)	Murayama and Uhlmann, 2015	N/A
Fission yeast Mis4-Ssl3	Murayama and Uhlmann, 2014	N/A
Fission yeast Mis4 N-191	Chao et al., 2015	N/A
Fission yeast Pds5	Murayama and Uhlmann, 2015	N/A
Fission yeast Wapl	Murayama and Uhlmann, 2015	N/A

**Critical Commercial Assays**		

SilverQuest Silver Staining Kit	ThermoFisher	Cat# LC6070
InFusion HD cloning kit	TaKaRa	Cat# 638910
Human IgG-Agarose	Sigma-Aldrich	Cat# A6284-5ML
Glutathione Sepharose 4B	GE Healthcare	Cat# 17075601
Ni-NTA Superflow (25 ml)	QIAGEN	Cat# 30410
HiTrap Heparin HP 1 ml	GE Healthcare	Cat# 17040601
Superdex 200 Increase 10/300 GL	GE Healthcare	Cat# 28990944
Superdex 75 Increase 10/300GL	GE Healthcare	Cat# 29148721
Superose 6, 10/300 GL	GE Healthcare	Cat# 17517201
Amicon Ultra-4 centrifuge filter unit	Sigma-Aldrich	Cat# UFC810096
Slide-A-Lyzer MINI Dialysis Devices, 20K MWCO	ThermoFisher	Cat# 69555
Dynabeads M-280 Streptavidin	ThermoFisher	Cat#11206D
Dynabeads Protein A	ThermoFisher	Cat#10002D
ECL Prime Western Blotting Detection Regent	GE Healthcare	Cat# RPN2232
Zeba Spin Desalting Columns, 7K MWCO, 0.5 mL	ThermoFisher	Cat# 89882
MICROSPIN S-400 HR, 50 COLUMNS	GE Healthcare	Cat#GE27-5140-01
TLC PEI Cellulose F	Merck	Cat#105725

**Experimental Models: Organisms/Strains**		

All yeast strains used in this study are listed in Table S1.	Lab stock and this study	N/A
*Escherichia coli*: BL21 (DE3) codonPlus RIPL chemical competent cells	Agilent Technologies	Cat#230280

**Oligonucleotides**		

TH1:[bioteg]agcgcagcgagtcagtgagcgagg	Sigma-Aldrich	N/A
TH2:cggtcgttcggctgcggcgagcgg	Sigma-Aldrich	N/A
TH3: [bioteg]cggtcgttcggctgcggcgagcgg	Sigma-Aldrich	N/A
TH4:agcgcagcgagtcagtgagcgagg	Sigma-Aldrich	N/A
TH5:cctttttacggttcctggcc	Sigma-Aldrich	N/A

**Recombinant DNA**		

pBluescript II KS(+)	Murayama and Uhlmann, 2014, 2015, Murayama et al., 2018	N/A
ssDNA of pBluescript II KS(+)	Murayama et al., 2018	N/A
M13KO7 Helper Phage	New England BioLabs	Cat# N0315S
JM109 competent cells	New England BioLabs	Cat#E4107
Plasmid: pMis4-PA	Murayama and Uhlmann, 2014	N/A
Plasmid: pMis4-N191-PA	Chao et al., 2015	N/A
Plasmid: pSsl3	Murayama and Uhlmann, 2014	N/A
Plasmid: pGEX-Wapl	Murayama and Uhlmann, 2015	N/A

**Software and Algorithms**		

Fiji ImageJ	open source	https://imagej.net/Fiji
UCSF ChimeraX	Resource for Biocomputing Visualization, and Informatics	https://www.cgl.ucsf.edu/chimerax/
PyMOL	Schrodinger	https://pymol.org/2/
PEAKS X+	Bioinfomatics Solutions Inc.	https://www.bioinfor.com/peaks-studio-x-plus/
xiVIEW	Rappsilber lab	https://xiview.org/xiNET_website/index.php
CCBuilder 2.0	open source	http://coiledcoils.chm.bris.ac.uk/ccbuilder2/builder
Clustal Omega	open source	https://www.ebi.ac.uk/Tools/msa/clustalo/

**Deposited Data**		

Protein-protein crosslink mass spectrometry (CLMS) data	PRIDE	PXD018608
DNA-protein crosslink mass spectrometry (DPC-MS)	PRIDE	PXD018600
Negative stain EM map	EMDB	EMD-10870
cryo-EM map	EMDB	EMD-10930
cryo-EM atomic coordinates	PDB	6YUF
Unprocessed gel images presented in this manuscript can be found at https://data.mendeley.com/datasets/9bddfnc7wb/draft?a=41d6ea5b-4cba-4f3e-b9f3-a42dfb09eff4	N/A	N/A

### Resource Availabilty

#### Lead Contact

Further information for resources and requests should be directed to and will be fulfilled by the Lead Contact, Frank Uhlmann (frank.uhlmann@crick.ac.uk).

#### Materials Availability

All reagents generated in this study are available from the Lead Contact without restriction.

#### Data and Code Availability

The negative stain map is available in EMDB, entry EMD-10870. The cryo-EM map and atomic coordinates are available in EMDB, entry EMD-10930 and PDB, entry 6YUF, respectively.

The CLMS data have been deposited to the ProteomeXchange Consortium via the PRIDE partner repository with the dataset identifier PXD018608.

The DPC-MS data have been deposited to the ProteomeXchange Consortium via the PRIDE partner repository with the dataset identifier PXD018600.

Unprocessed gel images presented in this manuscript can be found at https://data.mendeley.com/datasets/9bddfnc7wb/draft?a=41d6ea5b-4cba-4f3e-b9f3-a42dfb09eff4

### Experimental Model and Subject Details

#### Yeast Strains

All fission yeast cohesin tetramer complexes and Pds5 were expressed in W303 background budding yeast strains. Strains were cultured at 30°C in YP medium (2% peptone and 1% yeast extract) containing 2% raffinose until the optical density at 600 nm reached 1.0. Protein expression was induced by addition of 2% galactose for 4 h. Fission yeast Mis4-Ssl3 protein or Mis4-N191 protein was expressed in fission yeast strains. Fission yeast cells were cultured in EMM minimal medium supplemented with 30 μM thiamine at 30°C until the optical density at 595 nm reached 1.5, and protein expression was induced in EMM minimal medium lacking thiamine for 15 h. Genotypes of all strains used are listed in Table S1.

#### Bacteria

Fission yeast Wapl was expressed in the *E. coli* strain BL21-CodonPlus (DE3)-RIPL (Agilent Technologies). The genotype is: *E. coli B F- ompT hsdS(rB- mB-) dcm+ Tetr gal λ(DE3) endA Hte [argU proL Camr] [argU ileY leuW Strep/Specr]*.

### Method Details

#### Cloning of cohesin and its variants for protein purification

For construction of Head FRET wild-type and EQ-cohesin, SNAP-tag and CLIP-tag encoding sequences were fused to Psm1 C terminus and Psm3 C terminus in the shuttle vector YIplac211-Psm1/Psm3 or YIplac211-Psm1_E1161Q_ /Psm3_E1128Q_ that were constructed previously (Murayama and Uhlmann, 2014, 2015). The YIplac211-Psm1-SNAP/Psm3-CLIP vector and a YIplac128-Rad21/Psc3 expression vector were sequentially integrated into budding yeast at the *URA3* and *LEU2* loci, respectively.

For construction of the Kleisin-N FRET cohesin complex, SNAP-tag and CLIP-tag sequences were fused to Rad21 N terminus in the YIplac128-Rad21-Psc3 integration vector and Psm3 N terminus in the YIplac211-Psm1-Psm3 vector. Both vectors were integrated into budding yeast genome as before. Kleisin-N FRET Walker B motif mutant (EQ) and signature motif mutant (SQ) complexes were generated by site-directed mutagenesis on the YIplac211-Psm1/CLIP-Psm3 vector.

For construction of the kleisin circle construct, SNAP and CLIP-tag sequences were fused to Rad21 C terminus and N terminus in the YIplac128-Rad21-Psc3 integration vector. The first of the two separase recognition sequence in Rad21 was replaced with a tobacco etch virus (TEV) protease-recognition sequence. The YIplac128-CLIP-Rad21-SNAP/Psc3 expression vector was integrated into budding yeast harboring YIplac211-Psm1/Psm3.

For construction of SMC circle construct, SNAP-tag sequences were integrated into the Psm1 hinge region (between R593 and G594) and the Psm3 C terminus. CLIP-tag sequences were integrated into the Psm3 hinge region (between S631 and N632) and fused to the Psm1 C terminus in the YIplac211-Psm1/Psm3 vector. The expression vector was integrated into budding yeast harboring YIplac128-Rad21/Psc3.

For construction of N-terminally truncated N17-Rad21, a partial coding sequence (amino acids 18-646) was amplified by PCR, which replaced the full-length Rad21 gene in the YIplac128-Rad21/Psc3 vector by In-Fusion cloning. The YIplac128-N17-Rad21/Psc3 and YIplac211-Psm1-Psm3 vectors were integrated into budding yeast.

#### Protein expression, purification, labeling, and crosslinking

Fission yeast cohesin tetramer complexes including wild-type, walker B mutant (Psm1 E1161Q, Psm3 E1128Q, denoted as EQ-cohesin), Psm3 acetyl-acceptor site mutant, Rad21 N-terminal truncated mutant (Rad21 amino acids 18-646, denoted N17-cohesin), Kleisin-circle complex, SMC circle complex, Mis4-Ssl3, Mis4- N191(amino acids 192-1587), Pds5 and Wapl were expressed and purified following previously described methods (Chao et al., 2015; Murayama and Uhlmann, 2014, 2015)

All fission yeast cohesin complexes for FRET measurement (Head FRET wild-type and EQ-cohesin, Kleisin-N FRET wild-type, EQ and SG cohesin) were expressed and purified by sequential steps on IgG-Sepharose and heparin columns as described (Murayama and Uhlmann, 2014). The peak fractions from the heparin elution in R buffer (20 mM Tris/HCl, pH 7.5, 0.5 mM TCEP, 10% (v/v) glycerol) containing approximately 600 mM NaCl were concentrated to 500 μl by ultrafiltration. Cohesin was supplemented with 2 μM BG-surface Alexa 647, 1 mM DTT and 0.003% Tween20 and incubated at 25°C for 1 h. Now the labeling reaction was supplemented with 4 μM BC-surface Dy547 and incubated at 4°C for 16 h to complete the labeling. The labeled cohesin was applied to a Superose 6 10/300 GL gel filtration column that was developed in R buffer containing 200 mM NaCl and 0.003% Tween20. The peak fractions were concentrated to 500 μl by ultrafiltration.

To prepare head-crosslinked cohesin, Head FRET wild-type cohesin was expressed and purified by IgG-Sepharose chromatography as described above. Once loaded onto the heparin column, R buffer containing 100 mM NaCl and 4 μM SC-Cy5 crosslinker was injected and incubated at 25°C for 1 h, resulting mainly in SNAP tag coupling. After this incubation, the column was washed clear of crosslinker and heparin-bound cohesin was eluted and further incubated overnight at 4°C to allow CLIP tag coupling with SC-Cy5. The peak fractions of heparin purification step were concentrated to 500 μL by ultrafiltration and applied to a Superose 6 10/300 GL gel filtration column that was developed in R buffer containing 200 mM NaCl. The peak fractions were concentrated to 500 μL by ultrafiltration.

#### Topological cohesin loading assay

Topological cohesin loading onto DNA was performed in standard reactions (15 μl final volume) as previously described (Murayama and Uhlmann, 2014) with minor modifications. Cohesin (100 nM), Mis4-Ssl3 (100 nM) and pBluescript dsDNA were mixed on ice in reaction buffer (35 mM Tris-HCl pH 7.5, 0.5 mM TCEP, 25 mM NaCl, 1 mM MgCl_2_, 15% (w/v) glycerol and 0.003% (w/v) Tween 20). The reactions were initiated by addition of 0.5 mM ATP and incubated at 32°C for 120 min. The reactions were terminated by addition of 500 μL of ice-chilled Washing buffer A (35 mM Tris-HCl pH 7.5, 0.5 mM TCEP, 750 mM NaCl, 0.35% (w/v) Triton X-100. Anti-Pk antibody adsorbed to protein A conjugated magnetic beads was added to the terminated reactions and rocked at 4°C overnight. The beads were one time washed with Washing buffer A and three times with Washing buffer B (35 mM Tris-HCl pH 7.5, 0.5 mM TCEP, 500 mM NaCl and 0.1% (w/v) Triton X-100) and once with Washing buffer C (35 mM Tris-HCl pH 7.5, 0.5 mM TCEP, 50 mM NaCl and 0.1% (w/v) Triton X-100). The cohesin-bound DNA was eluted in 15 μl of elution buffer (10 mM Tris-HCl pH 7.5, 1 mM EDTA, 50 mM NaCl, 0.75% SDS and 1 mg/ml protease K) by incubation at 50°C for 20 min. The recovered DNA was separated by 0.8% agarose gel electrophoresis in TAE buffer and stained with SYBR gold. Gel images were captured using a Typhoon FLA 9500 biomolecular imager and band intensities quantified using ImageJ.

#### Bulk FRET measurement

All fluorescence measurements were carried out at room temperature in reaction buffer (35 mM Tris-HCl pH 7.5, 0.5 mM TCEP, 25 mM NaCl, 1 mM MgCl_2_, 15% (w/v) glycerol and 0.003% (w/v) Tween 20). 40 μL of reaction mixtures containing 10 nM Dy547 and Alexa 647-labeled cohesin, 100 nM Mis4-Ssl3 and 3 nM DNA substrate were mixed and the reaction was started by addition of 0.5 mM ATP. Alternatively, 0.5 mM ADP or 0.5 mM ADP and 0.5 mM BeF_2_, 0.5 mM BeSO_4_ + 10 mM NaF, 0.5 mM AlCl_3_ + 10 mM NaF, or 0.5 mM Na_3_VO_4_ were included instead of ATP. The reactions were incubated at 32°C for 20 min. The samples were applied to a 384-well plate and fluorescence spectra of the cohesin complex were collected on a CLARIOstar high performance plate reader. Samples were excited at 525 nm and emitted light was recorded between 560 - 700 nm in 0.5 nm increments. To evaluate FRET changes caused by cohesin’s conformational changes across different experimental conditions, we report relative FRET efficiency, I_A_/(I_D_ + I_A_), where I_D_ is the donor emission signal intensity at 565 nm resulting from donor excitation at 525 nm and I_A_ is the acceptor emission signal intensity at 665 nm resulting from donor excitation at 525 nm.

#### DNA gripping experiments

For DNA gripping analyses, we immobilized DNA on magnetic beads. A 3 kb linear DNA substrate was prepared by PCR amplification with 5′-biotinylated oligonucleotide TH1 and unmodified TH2 using pBluescript dsDNA as the template. The 3 kb DNA loop substrate was made by PCR amplification with a pair of both 5′-biotinylated oligonucleotides TH1 and TH3 using pBluescript dsDNA as the template. Streptavidin conjugated magnetic beads were washed with DNA binding buffer, DBB (10 mM Tris-HCl pH 7.5, 2 M NaCl, 1 mM EDTA, 0.03% Tween20) and resuspended in 2 volumes of DBB. 100 ng biotin-labeled DNA was mixed with 20 μL beads and incubated at room temperature for 1 h. Beads were washed 3 times with reaction buffer (35 mM Tris-HCl pH 7.5, 0.5 mM TCEP, 25 mM NaCl, 1 mM MgCl_2_, 15% (w/v) glycerol and 0.003% (w/v) Tween 20) and resuspended in reaction buffer supplemented with 1 mg/ml BSA and 2.5 mU poly-dIdC:dIdC. After 30 min incubation, DNA-beads were washed 3 times with reaction buffer. The standard reaction volume was 15 μl, containing 100 nM cohesin, 100 nM Mis4-Ssl3, 100 nM Mis4-N191, 100 nM Pds5 and 100 nM Wapl in reaction buffer. The reaction mixture was added to the DNA beads (containing 3.3 nM dsDNA molecules) on ice. The reactions were started by addition of 0.5 mM ATP, or 0.5 mM ADP and 0.5 mM BeSO_4_ + 10 mM NaF, and incubated at 32°C for 20 min. After the incubation, beads were washed three times with Washing buffer C (35 mM Tris-HCl pH 7.5, 0.5 mM TCEP, 50 mM NaCl and 0.1% (w/v) Triton X-100) or Washing buffer D (35 mM Tris-HCl pH 7.5, 0.5 mM TCEP, 135 mM NaCl and 0.1% (w/v) Triton X-100) and once with Washing buffer C. The beads were divided into two for detection of protein and DNA. Protein samples were eluted with SDS-sample buffer (50 mM Tris-HCl pH 6.8, 2% SDS, 10% Glycerol, 50 mM DTT, 0.02% Bromophenol Blue) and boiled for 5 min. The DNA sample was eluted in buffer containing 3 mM biotin and incubated overnight at room temperature. DNA-bound proteins were separated by SDS-PAGE and analyzed by immunoblotting using the indicated antibodies. The recovered DNA was analyzed by 0.8% agarose gel electrophoresis as described above.

#### EM sample preparation of cohesin in the gripping state

For EM sample preparation, we used a 125 bp linear dsDNA substrate that was generated by PCR amplification with a pair of oligonucleotides TH1 and TH5 using pBluescript dsDNA as the template. 200 nM cohesin, 200 nM Mis4-Ssl3, 200 nM 125bp dsDNA were mixed in reaction buffer on ice. The reaction was started by addition of 0.5 mM ADP and 0.5 mM BeSO_4_ + 10 mM NaF and incubated at 32°C for 20 min. After incubation, an equal volume of 2 x Washing buffer D (35 mM Tris-HCl pH 7.5, 0.5 mM TCEP, 135 mM NaCl and 0.1% (w/v) Triton X-100) was added for further incubation at 4°C for 10 min. The reaction mixture of a total volume of 50 μL was loaded onto 20–50% (weight/volume) linear sucrose gradients prepared in EM buffer (20 mM HEPES-KOH pH 7.5, 25 mM NaCl, 0.5 mM TCEP). Centrifugation was in a MLS-50 rotor (Beckman) at 37,000 rpm for 16 h at 4°C. 50 μL fractions were collected from top to bottom and protein and DNA in each fraction were analyzed by SDS-PAGE followed by silver-staining or agarose gel electrophoresis to identify peak fractions containing the cohesin-loader-DNA complex. Sucrose in the peak fractions was removed by passing three times through spin desalting columns before application to EM grids.

#### Negative stain EM data acquisition and image processing

A 300-mesh, continuous carbon copper grid (EM Resolutions, C300Cu100) was glow-discharged at 45 mA for 30 s. A 4 μL sample was applied and incubated for 1 min, followed by blotting of excess volume and grid staining in four 50 μL droplets of 2% uranyl acetate for 5, 10, 15, 20 s respectively. The grid was subsequently blotted dry. Micrographs were collected at x30,000 nominal magnification (3.45 Å pixel size) with a defocus range of −0.5 to −2.5 μm using a FEI Tecnai LaB6 G2 Spirit electron microscope operated at 120 kV and equipped with a 2K x 2K GATAN UltraScan 1000 CCD camera.

Contrast transfer function parameters were estimated using Gctf v1.06 (Zhang, 2016) and particles were picked semi-automatically with e2boxer in EMAN2 v2.07 (Tang et al., 2007). Subsequent image processing was performed in RELION v3.0.4 (Zivanov et al., 2018). Particles were initially extracted with a box size of 128 pixels and sorted by reference-free 2D classification with CTF-correction using the additional argument --only_flip_phases. To allow visualization of extended Psm1-Psm3 coiled coils, selected cohesin particles were re-extracted with a box size of 192 pixels and processed through one additional round of 2D classification. A reference-free initial 3D model was also created in RELION and used as an input for 3D refinement using particles with the larger box size.

#### Cryo-EM data acquisition and image processing

A 400-mesh lacey copper grid with a layer of ultra-thin carbon (Agar Scientific) was glow-discharged at 45 mA for 1 min. A 4 μL sample was applied and incubated for 2 min, followed by blotting of excess volume for 0.5 s using a Vitrobot Mark IV (FEI ThermoFisher) operated at room temperature and 100% humidity. To increase particle concentration, two additional 4 μL samples were applied to the grid for 2 min each, with 0.5 s blotting in between. After a final blot of 3 s the grid was plunge-frozen into liquid ethane. High-resolution cryo-EM data were acquired on a FEI Titan Krios electron microscope operated at 300 kV and equipped with Falcon 3EC Direct Electron Detector. Micrographs were collected at x75,000 nominal magnification (1.09 Å pixel size) as 30-frame movies with a total electron dose of 33.8 e^-^/Å^2^ and a defocus range of −2.0 to −4.0 μm. A second dataset was collected with a phase plate using a GATAN K2 Summit direct electron detector operated in counting mode. Micrographs were collected at x130,000 nominal magnification (1.09 Å pixel size) as 40-frame movies with a total electron dose of 49 e^-^/Å^2^ and −0.5 μm defocus.

For the first dataset (no phase-plate), 30-frame movies were corrected for beam-induced movement using 5 × 5 patch alignment with all frames in MotionCor2 (Zheng et al., 2017). Contrast transfer function parameters were estimated on non-dose-weighted micrographs using Gctf v1.06 and particles were picked with crYOLO (Wagner et al., 2019). Subsequent image processing was performed in RELION v3.0.4 and cryoSPARC v2.14.2. Initially 883,184 particles were extracted from 12,085 micrographs in RELION using a box size of 360 pixels. After reference-free 2D classification in cryoSPARC 792,173 cohesin particles were selected and utilized to reconstruct an ab-initio 3D model, which was subsequently used as a starting model for non-uniform refinement. Following 3D refinement in RELION the particle subset was subjected to two rounds of 3D classification using a mask encompassing the cohesin core only. Ultimately 255,148 particles were selected and refined in cryoSPARC using non-uniform refinement followed by local non-uniform refinement of the cohesin core, resulting in a structure at 3.9 Å resolution. The final half-maps were used to produce a density modified map using the Phenix’s tool ResolveCryoEM (Terwilliger et al., 2020). This map showed significant improvements in side chain density and overall interpretability.

For the second dataset (phase-plate), 40-frame movies were corrected for beam-induced movement using 5 × 5 patch alignment using all frames in MotionCor2. Contrast transfer function parameters were estimated on non-dose-weighted micrographs using CTFFIND v4.1.10 (Rohou and Grigorieff, 2015) and particles were picked with crYOLO. Subsequent image processing was performed in RELION v3.0.4 and cryoSPARC v2.14.2. Initially 330,024 binned-by-2 particles were extracted from 5,972 micrographs in RELION using a box size of 276 pixels (2.18 Å/pixel). After reference-free 2D classification in cryoSPARC 227,159 cohesin particles were selected and utilized to reconstruct an ab-initio 3D model, which was subsequently used as a starting model for non-uniform refinement. The particles were re-extracted with a smaller box size of 180 pixels (2.18 Å/pixel) and 3D refined in RELION, which revealed a flexible element connected to the cohesin core. To further characterize this peripheral element, particles were 3D-classified without image alignment using a mask encompassing only this density region. 80,325 particles were selected and 3D-autorefined in RELION without a mask. To increase the resolution of the flexible element and assess the conformational changes sampled in the cohesin complex, multibody refinement (Nakane et al., 2018) was performed using masks encompassing either the core or the flexible region. The two signal-subtracted particle stacks generated during multibody refinement in RELION were then exported for non-uniform refinement in cryoSPARC. Although the flexible region could not be resolved to subnanometer resolution, a defined, rigid body could be identified, into which a homology model of Psc3 could be unambiguously docked using the Fit-in map command in UCSF Chimera (Pettersen et al., 2004). The homology model was generated with SWISS-MODEL (Waterhouse et al., 2018) and based on PDB entry 6H8Q (Li et al., 2018). See also Table S2 for the image processing workflow for the cryo-EM core structure as well as the multibody refinement workflow that led to the Identification of a separate rigid body identified as Psc3.

#### Model building and validation

SWISS-MODEL was used to obtain homology models for Psm1, Psm3, Rad21 (PDB entries 4UX3 and 1W1W) (Gligoris et al., 2014; Haering et al., 2004), and Mis4, PDB entry 5T8V (Kikuchi et al., 2016). These models were docked into the cryo-EM map using the Fit in Map command in USCF Chimera (Pettersen et al., 2004). These models were refined against the map using Namdinator (Kidmose et al., 2019) and the resulting model was used as a starting point for manual adjustments in Coot (Emsley et al., 2010). The resulting model was then subjected to an iterative process of real space-refinement using Phenix.real_space_refinement (Adams et al., 2010) with geometry and secondary structure restraints followed by manual inspection and adjustments in Coot. Residues 552-583 from Rad21(chain B) and 209-302 from Mis4 (chain D) were docked into the map by rigid-body fitting of the corresponding homology models. The geometries of the atomic model were evaluated by MolProbity (Williams et al., 2018). Cryo-EM data acquisition, 3D reconstruction information and atomic model refinement statistics are summarized in Table S3. Figures were prepared with UCSF Chimera and ChimeraX (Goddard et al., 2018).

#### SDA-based protein-protein crosslink mass spectrometry (CLMS) analysis

##### Sample preparation

Protein crosslinking of the cohesin complex was performed in two conditions. An initial state contained all components except nucleotide. The DNA gripping state was achieved by addition of ADP and BeSO_4_ + NaF. All materials (cohesin, loader and DNA) were dialyzed in SDA crosslinking buffer (35 mM HEPES-KOH pH 7.5, 0.5 mM TCEP, 25 mM NaCl, 1 mM MgCl_2_, 15% (w/v) glycerol and 0.003% (w/v) Tween 20) at 4°C for 3 h. Cohesin (200 nM), Mis4-Ssl3 (200 nM) and 125 bp dsDNA (200 nM) were mixed on ice in SDA crosslinking buffer. The reaction in each condition was started in the absence of nucleotide or in the presence of 0.5 mM ADP and 0.5 mM BeSO_4_ + 10 mM NaF at 32°C. After 20 min incubation, SDA was added to 50 μg of the cohesin complex at increasing crosslinker weight ratios. (Protein: SDA = 1:1.3, 1:1.9 and 1: 3.8). The diazirine group in SDA was photo-activated using UV irradiation at 365 nm from an ultraviolet crosslinker (Spectrum). Samples were mounted in a 96-well plate, placed on ice at a distance of 5 cm from the UV-A lamp and irradiated for 20 min. After UV irradiation, the sample was further incubated on ice for 2 h to allow further time for NHS crosslinking. The reaction mixtures from the three protein: crosslinker ratios were combined and quenched with 50 mM ammonium bicarbonate. 4 sample volumes of cold acetone were added and incubated at −20°C for 1 h. Precipitated proteins were collected by centrifugation and dried in a vacuum concentrator.

##### CLMS sample analysis

Both samples were resolubilized in 100 μl digestion buffer (8M urea in 100 mM ammonium bicarbonate) to an estimated protein concentration of 1 mg/ml. Dissolved protein sample was reduced by addition of 0.5 μL 1M dithiothreitol (DTT) at room temperature for 30 min. The free sulfhydryl groups in the sample were then alkylated by adding 3 μl 500 mM iodoacetamide and incubation at room temperature for 20 min in the dark. After alkylation, 0.5 μL 1M DTT was added to quench excess of iodoacetamide. Next, protein samples were digested with LysC (at a 50:1 (m/m) protein to protease ratio) at room temperature for four h. The sample was then diluted with 100 mM ammonium bicarbonate to reach a urea concentration of 1.5 M. Trypsin was added at a 50:1 (m/m) protein to protease ratio to further digest proteins overnight (~15 h) at room temperature. Resulting peptides were desalted using C18 StageTips (Rappsilber et al., 2007).

For each sample, resulting peptides were fractionated using size exclusion chromatography in order to enrich for crosslinked peptides (Leitner et al., 2014). Peptides were separated using a Superdex Peptide 3.2/300 column (GE Healthcare) at a flow rate of 10 μl/minute. The mobile phase consisted of 30% (v/v) acetonitrile and 0.1% trifluoroacetic acid. The earliest six peptide-containing fractions (50 μL each) were collected. Solvent was removed using a vacuum concentrator. The fractions were then analyzed by LC-MS/MS.

LC-MS/MS analysis was performed using an Orbitrap Fusion Lumos Tribrid mass spectrometer (Thermo Fisher Scientific), connected to an Ultimate 3000 RSLCnano system (Thermo Fisher Scientific). Each size exclusion chromatography fraction was resuspended in 1.6% v/v acetonitrile 0.1% v/v formic acid and analyzed with replicated LC-MS/MS acquisitions. Peptides were injected onto a 50 cm EASY-Spray C18 LC column (Thermo Scientific) that is operated at 50°C column temperature. Mobile phase A consists of water, 0.1% v/v formic acid and mobile phase B consists of 80% v/v acetonitrile and 0.1% v/v formic acid. Peptides were loaded and separated at a flowrate of 0.3 μl/min. Peptides were separated by applying a gradient ranging from 2% to 45% B over 90 min. The gradient was optimized for each fraction. Following the separating gradient, the content of B was ramped to 55% and 95% within 2.5 min each. Eluted peptides were ionized by an EASY-Spray source (Thermo Scientific) and introduced directly into the mass spectrometer.

The MS data were acquired in the data-dependent mode with the top-speed option. For each three-second acquisition cycle, the full scan mass spectrum was recorded in the Orbitrap with a resolution of 120,000. The ions with a charge state from 3+ to 7+ were isolated and fragmented using higher-energy collisional dissociation (HCD). For each isolated precursor, one of three collision energy settings (26%, 28% or 30%) was selected for fragmentation using a data-dependent decision tree based on the m/z and charge of the precursor. The fragmentation spectra were then recorded in the Orbitrap with a resolution of 50,000. Dynamic exclusion was enabled with single repeat count and 60 s exclusion duration.

MS2 peak lists were generated from the raw mass spectrometric data files using the MSConvert module in ProteoWizard (version 3.0.11729). The default parameters were applied, except that Top MS/MS Peaks per 100 Da was set to 20 and the denoising function was enabled. Precursor and fragment m/z values were recalibrated. Identification of crosslinked peptides was carried out using xiSEARCH software (https://www.rappsilberlab.org/software/xisearch) (version 1.7.0) (Mendes et al., 2019). The “initial state” and “gripping state” samples were processed separately. For each sample, peak lists from all LC-MS/MS acquisitions were searched against the sequence and the reversed sequence of cohesin and loader subunits (Psm1, Psm3, Rad21, Psc3, Mis4 and Ssl3). The following parameters were applied for the search: MS accuracy = 4 ppm; MS2 accuracy = 10 ppm; enzyme = trypsin (with full tryptic specificity); allowed number of missed cleavages = 2; missing monoisotopic peak = 2; crosslinker = SDA (the reaction specificity for SDA was assumed to be for lysine, serine, threonine, tyrosine, and protein N-termini on the NHS ester end and any amino acids for the diazirine end); fixed modifications = carbamidomethylation on cysteine; variable modifications = oxidation on methionine and SDA loop link. Identified crosslinked peptide candidates were filtered using xiFDR (Fischer and Rappsilber, 2017). A false discovery rate of 1% on residue-pair level was applied with “boost between” option selected. A list of identified crosslinked residue pairs is reported in Table S4.

#### DNA-protein crosslink mass spectrometry (DPC-MS) analysis

##### Sample preparation

For DNA-protein crosslinking, we prepared two types of dsDNA probes. A 125 bp linear dsDNA was amplified by PCR with 5′-biotinylated oligonucleotide TH1 and non-modified oligonucleotide TH5 using pBluescript dsDNA as the template. PCR reaction mixtures contained 5 ng/ml template DNA, 0.3 μM of each primer, 0.2 mM each of dATP, dCTP and dGTP, 0.02 mM dTTP, 0.18 mM aminoallyl-dUTP and 0.025 unit/μl Go-taq DNA polymerase in 1x Go-taq buffer (Promega). A 3 kb circular dsDNA was prepared by primer extension on single stranded DNA. 5′-biotinylated TH1 oligonucleotide primer was annealed to single strand DNA templates of pBluescript, prepared using M13KO7 helper phage (Murayama et al., 2018). For second strand synthesis, the primer-template mix was incubated in 20 mM Tris-HCl (pH 7.5), 10 mM MgCl_2_, 1 mM DTT, 0.4 mM each of dATP, dCTP, dGTP and aminoallyl-dUTP, 0.1 mg/ml BSA and 0.04 unit/μl T7 DNA polymerase (New England Biolabs) at 37°C for 3 h. After synthesis, the buffer of the DNA samples was exchanged to SDAD crosslinking buffer (100 mM NaHCO_3_ pH 8.3) using MicroSpin S400 columns (GE Healthcare). 1 μg of dsDNA was incubated with 2 mM SDAD crosslinker in 25 μL of SDAD crosslinking buffer at 25°C overnight. The diazirin-decorated dsDNA (SDAD-DNA) probe was dialyzed in DNA dialysis buffer (10 mM Tris-HCl pH 7.5, 0.1 mM EDTA).

For DNA-protein crosslinking 200 nM cohesin, 200 nM Mis4-Ssl3, 20 ng/μl linear 125 bp or circular 3 kb SDAD-DNA probe were mixed in reaction buffer, and the DNA gripping reaction was initiated by addition of 0.5 mM ADP and 0.5 mM BeSO_4_ + 10 mM NaF at 32°C for 30 min. An equal volume of 2 x Washing buffer D (35 mM Tris-HCl pH 7.5, 0.5 mM TCEP, 135 mM NaCl and 0.1% (w/v) Triton X-100) was added to the reaction mixture and incubated at 4°C for 10 min. The sample was mounted on a 96-well plate, placed on ice at a distance of 5 cm from the UV-A lamp and irradiated for 10 min, as described above for SDA protein-protein crosslinking. After UV irradiation, the buffer of the samples was exchanged with Protease buffer (100 mM ammonium bicarbonate pH 8.0) using MicroSpin S400 columns. Lys-C protease was added (1:20 (m/m) protease to protein ratio) and incubated at 37°C overnight. To remove non-crosslinked peptide from the DNA, an equal volume of 2 x RIPA buffer (100 mM Tris-HCl pH 8, 100 mM NaCl, 0.2% SDS) was added to the sample, followed by incubation at 50°C for 30 min. The DNA with crosslinked peptides was purified by Superdex75 size exclusion chromatography developed with 20 mM Tris-HCl pH 7.5, 200 mM NaCl. The recovered DNA-peptide complexes in the void fraction were supplemented with NaCl to 1 M final concentration and 0.1% (w/v) Tween-20. The biotinylated DNA was recovered using streptavidin M280 magnetic beads (Invitrogen) at 25°C for 1 h. DNA-beads were washed three times with 1 x RIPA buffer and five times with peptide elution buffer (20 mM Tris-HCl pH 7.5, 200 mM NaCl). DNA-crosslinked peptides were now eluted by addition of peptide elution buffer containing 25 mM DTT and incubation at 37°C for 30 min.

In the experiment comparing cohesin’s initial binding state and the gripping state, the incubation and crosslinking were performed as above without or with 0.5 mM BeSO_4_ + 10 mM NaF. After the 32°C incubation, the sample was directly mounted on a 96-well plate without washing buffer addition and the plate was UV irradiated on ice for 10 min. The irradiated sample was then treated as described above.

##### DPC-MS sample analysis

Peptide solutions in the DTT peptide elution buffer were transferred into Total Recovery vials (Waters) for injection without further clean-up or concentration. Samples were analyzed by online nanoflow LC-MS/MS using an Orbitrap Fusion Lumos mass spectrometer (Thermo Scientific) coupled to an Ultimate 3000 RSLCnano (Thermo Scientific). 15 μl of sample was loaded via autosampler into a 20 μl sample loop and pre-concentrated onto an Acclaim PepMap 100 75 μm x 2 cm nanoviper trap column with loading buffer, 2% v/v acetonitrile, 0.05% v/v trifluoroacetic acid, 97.95% water (Optima grade, Fisher Scientific) at a flow rate of 7 μl/min for 6 min in the column oven held at 40°C. Peptides were gradient eluted and separated with a C_18_ 75 μm x 50 cm, 2 μm particle size, 100 Å pore size, reversed phase EASY-Spray analytical column (Thermo Scientific) at a flow rate of 275 nl/min and with the column temperature held at 40°C, with a spray voltage of 2100 V using the EASY-Spray Source (Thermo Scientific). Gradient elution buffers were A 0.1% v/v formic acid, 5% v/v DMSO, 94.9% v/v water and B 0.1% v/v formic acid, 5% v/v DMSO, 20% v/v water, 74.9% v/v acetonitrile (all Optima grade, Fisher Scientific aside from DMSO, Honeywell Research Chemicals). The gradient elution profile used was 8% B to 40% B over 60 min.

The instrument method used an MS1 Orbitrap scan resolution of 120,000 at FWHM m/z 200, quadrupole isolation, mass range 375-1500 m/z, RF Lens 40%, AGC target 4e5, maximum injection time 50 ms and spectra were acquired in profile. Monoisotopic Peak Determination was set to the peptide mode, and only precursors with charge states 2-6 were permitted for selection for fragmentation. Dynamic Exclusion was enabled to exclude after n = 1 times for 20 s with high and low ppm mass tolerances of 10 ppm. MS2 scans were acquired in the ion trap following HCD fragmentation with fixed collision energy of 32% and was performed on all selected precursor masses using a cycle time based on data-dependent mode of acquisition set to 3 s. The parameters used for the HCD MS2 scan were quadrupole isolation with an isolation window width of 1.2 m/z, first mass 110 m/z, AGC target 2e3, maximum injection time 300 ms and the scan data were acquired in centroid mode at the rapid scan rate.

A FASTA database containing only the sequences of the six subunits of the cohesin complex and loader was used for the PEAKS search conducted within PEAKS Studio (Bioinformatics Solutions Inc). A modification corresponding to the diazirine moiety after reduction (C_7_H_13_NOS, 159.07178) was created and considered as a variable modification along with the oxidation of methionine residues. Other parameters of the search were digestion enzyme LysC with a maximum of 2 missed cleavages, peptide mass tolerance 5ppm and fragment mass tolerance 0.6 Da.

#### SC-Cy5 crosslinking experiments

The SC-Cy5 crosslinker was synthesized as previously described (Gautier et al., 2009). Using crosslinkable cohesin complexes (kleisin-circle or SMC-circle) and a DNA-loop substrate, we performed DNA gripping assay as described above. Following the DNA gripping reaction, DNA-beads were washed 3 times with Washing buffer D (35 mM Tris-HCl pH 7.5, 0.5 mM TCEP, 135 mM NaCl and 0.1% (w/v) Triton X-100), and supplemented with 4 μM SC-Cy5 and 1 mM DTT in Washing buffer D. Crosslinking was carried out at 32°C for 60 min. DNA-beads were then divided into three parts. One part was immediately eluted with SDS sample buffer containing 3 mM biotin and served as the input sample. The second sample was washed 5 times with Washing buffer D. The third sample was washed 5 times with SDS buffer (35 mM Tris-HCl pH 7.5, 0.5 mM TCEP, 100 mM NaCl and 0.1% SDS). The second and third samples were then supplemented with SDS sample buffer containing 3 mM biotin and boiled for 10 min to elute DNA and protein. Samples were analyzed by SDS-PAGE, followed by in-gel detection of Cy5 or immunoblotting with indicated antibodies.

To further evaluate topological DNA entrapment by the circularized kleisin, beads following SDS washes were divided into two, equilibrated with DNA digestion buffer (35 mM Tris-HCl pH 7.5, 0.5 mM TCEP, 100 mM NaCl, 10 mM MgCl_2_, 0.1 mg/ml BSA, 0.1% Triton X-100) and treated without or with 1 U/μl restriction enzyme PstI in DNA digestion buffer. Alternatively, the sample was equilibrated with TEV digestion buffer (50 mM Tris-HCl pH 8.0, 100 mM NaCl, 0.5 mM EDTA, 1 mM DTT) and treated without or with 0.25 U/μl TEV protease in TEV digestion buffer. After a 20 min incubation at 32°C, the beads and supernatant fractions were separated, SDS sample buffer containing 3 mM biotin added to each and samples boiled for 10 min.

#### ATPase assay

Cohesin (150 nM) and Mis4-Ssl3 (100 nM) were mixed with pBluescript dsDNA in reaction buffer (15 μL in final volume). The reactions were initiated by addition of 0.25 mM ATP, spiked with [γ-33P]-ATP, and incubated at 32°C. Aliquots (2 μl) were taken after 0, 15, 30, and 60 min and terminated by addition of 6 μL of 0.5 M EDTA pH 8.0. The products were separated by thin layer chromatography on TCL polyethylenimine cellulose F sheets (Merck), developed with 400 mM LiCl in 1 M formic acid. Plates were analyzed using a Typhoon FLA 9500 Phosphor-imager (GE Healthcare).

### Quantification and Statistical Analysis

#### Bulk FRET analysis

The fluorescent signals of proteins were detected using a CLARIOstar high performance plate reader and the FRET efficiency calculated as described in Method Details. The experiments were repeated at least three times. The results from all individual experiments are shown, together with their means and standard deviations.

#### Cohesin loading and DNA gripping experiments

Immunoblots were developed using ECL reagents (GE Healthcare). The chemiluminescent signals were detected using an Amersham Imager 600 (GE Healthcare) or Amersham Hyperfilm ECL (GE Healthcare). In-gel fluorescent signals of labeled proteins were detected using a Typhoon FLA9500 imager (GE Healthcare). Recovered DNAs were separated by agarose-gel electrophoresis and stained by SYBR gold. The DNA signals were also detected by the Typhoon FLA9500 imager and signal intensities were quantified using ImageJ software. The graphs depict means and the error bars represent standard deviations from three independent experiments.

#### ATPase Assay

Reaction products containing radioisotope were separated by thin layer chromatography and quantified using the Typhoon FLA9500 imager. The signal intensities were quantified in ImageJ. The graphs depict means and the error bars represent standard deviations from three independent experiments.

## Supporting Citations

The following reference appears in the Supplemental Information: Haering et al. (2002).

## Supplemental Information

Document S1. Figures S1–S7

Table S1. Yeast Strains Used in this Study, Related to STAR Methods

Table S2. Image Processing Workflow for the Cryo-EM Core Structure as well as the Multibody Refinement Workflow that Led to Identification of a Separate Rigid Body Identified as Psc3, Related to STAR Methods

Table S3. Cryo-EM Data Collection, Refinement, and Validation Statistics, Related to STAR Methods

Table S4. List of Identified Crosslinked Residue Pairs, Related to STAR Methods

Video S1. DNA Entry into the Cohesin Ring

Document S2. Article plus Supplemental Information
