# Supplementary material for: A Structure-Based Mechanism for DNA Entry into the Cohesin Ring
Source: Mol Cell. 2020 Sep 17;79(6):917–933.e9. doi: 10.1016/j.molcel.2020.07.013 (PMC7507959; doi:10.1016/j.molcel.2020.07.013)
Supplement: Document S1. Figures S1–S7 [file mmc1.pdf]

Molecular Cell, Volume *79*

Supplemental Information

A Structure-Based Mechanism

for DNA Entry into the Cohesin Ring

Torahiko L. Higashi, Patrik Eickhoff, Joana S. Sousa, Julia Locke, Andrea Nans, Helen R. Flynn, Ambrosius P. Snijders, George Papageorgiou, Nicola O'Reilly, Zhuo A. Chen, Francis J. O'Reilly, Juri Rappsilber, Alessandro Costa, and Frank Uhlmann

Supplemental Figure 1

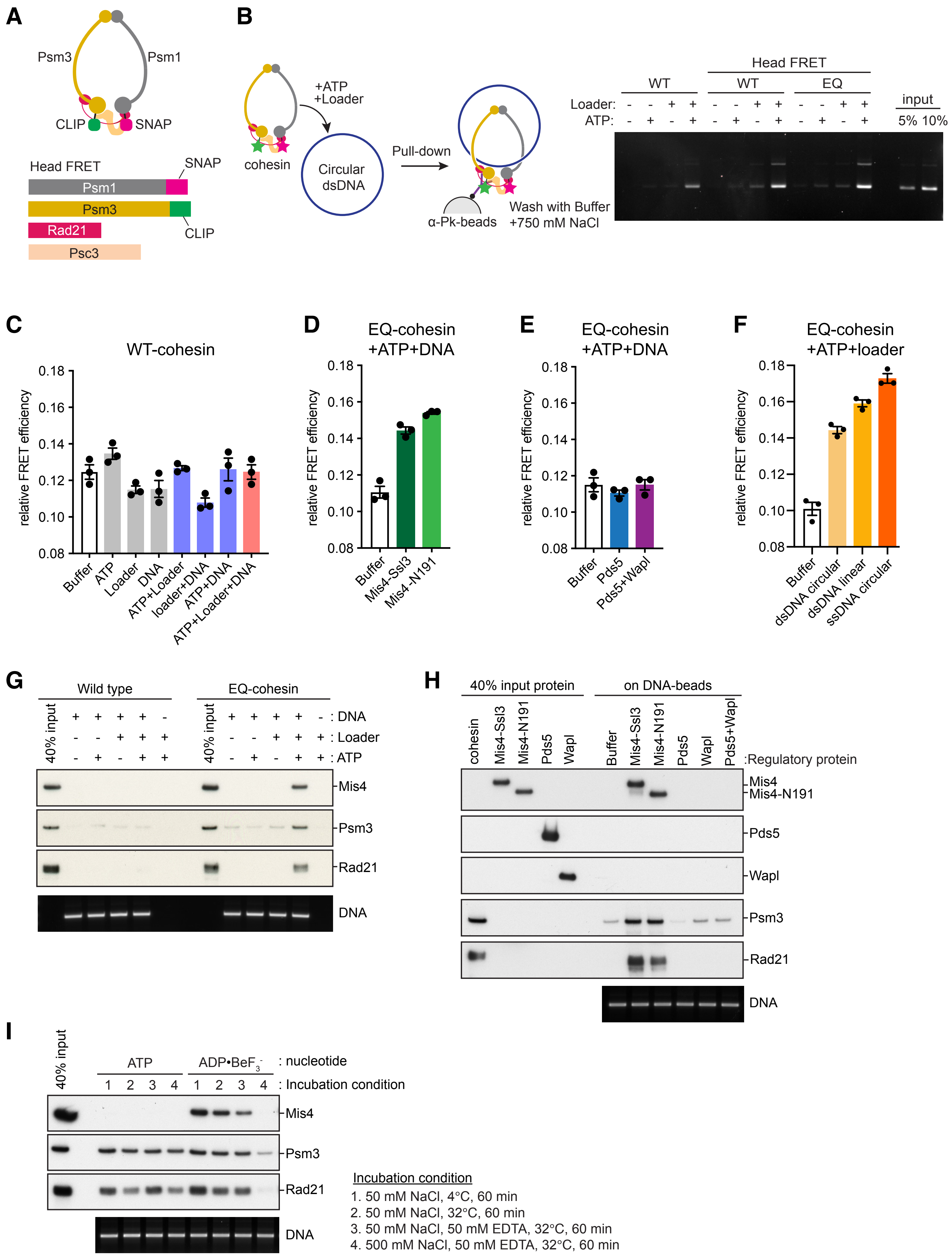

Figure S1. Characterization of Cohesin's DNA Gripping State, Related to Figure 1

(A) Schematic of the modified cohesin complex to record FRET between the ATPase heads. Psm1 and Psm3 were fused to a C-terminal SNAP- and CLIP-tag, respectively.

(B) Schematic of a cohesin loading reaction and an experiment to compare the performance of untagged cohesin (WT) with head-labeled wild type (Head FRET, WT) and Walker B mutant cohesin (Head FRET, EQ). Following incubation, cohesin was retrieved by immunoprecipitation, washed with buffer containing 500 mM NaCl, and the retained DNA was analyzed by agarose gel electrophoresis.

(C) Head FRET efficiencies of wild type cohesin in absence or presence of combinations of ATP, loader and a 3 kb circular plasmid DNA.

(D) Ability of the Mis4-Ssl3 cohesin loader complex, or Mis4-N191, to augment head FRET efficiency of EQ-cohesin in the presence of ATP and a 3 kb circular plasmid DNA.

(E) Effect of the cohesin unloading factors Pds5 or Pds5-Wapl on head FRET efficiency of EQ-cohesin in the presence of ATP and a 3 kb circular plasmid DNA.

(F) 3 kb circular plasmid dsDNA was compared to linear 3 kb dsDNA and a 3 kb circular ssDNA for its ability to stimulate head FRET efficiency of EQ-cohesin in the presence of ATP and loader. Results from three independent repeats of the experiment, their means and standard deviations are shown in (C) – (F).

(G) DNA gripping experiment as in Figure 1D, but comparing wild type and EQ-cohesin in the presence of ATP and additional indicated components.

(H) Comparison of the ability of Mis4-Ssl3, Mis4-N191, Pds5 and Pds5-Wapl to promote DNA gripping state formation in an experiment performed with wild type cohesin in the presence of ADP·BeF_3^-.

(I) Comparison of cohesin's DNA binding stability following incubation with loader and either hydrolyzable ATP or non-hydrolyzable ADP·BeF_3^- using a bead-bound DNA loop substrate. Following the loading reaction and an initial wash with cold buffer containing 500 mM NaCl, the beads were subjected to a 60 minute incubation under one of the four different indicated washing conditions. Beads were then recovered and bound DNA and protein analyzed.

Supplemental Figure 2

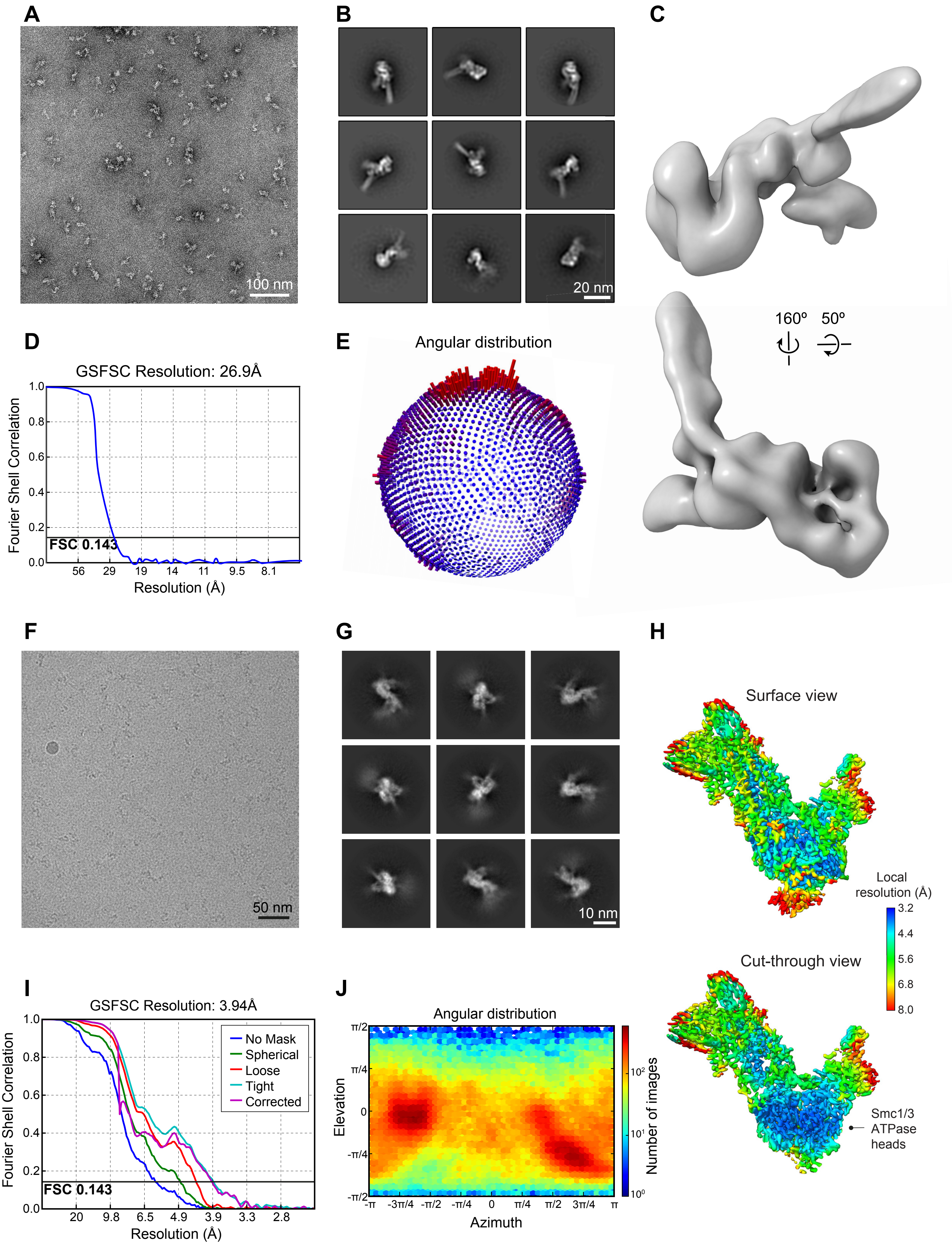

Figure S2. Negative Staining and cryo-EM Data Collection and Image Processing, Related to Figure 2

(A) Representative negative stain micrograph.

(B) Negative stain 2D class averages.

(C) Negative stain 3D reconstruction.

(D) Fourier Shell correlation plot with resolution indicated according to the 0.143 criterion.

(E) Angular distribution as depicted in RELION.

(F) Aligned cryo-EM movie sum obtained from frames collected on a Falcon 3 direct electron detector.

(G) Cryo-EM 2D averages.

(H) Surface rendering and cut-through view of the cryo-EM cohesin core reconstruction colored according to the local resolution.

(I) Fourier Shell correlation plot with resolution indicated according to the 0.143 criterion. The graph was generated using cryoSPARC.

(J) Angular distribution.

See also Table S2 for the image processing workflow for the cryo-EM core structure as well as the multibody refinement workflow that led to the identification of a separate rigid body identified as Psc3.

Supplemental Figure 3

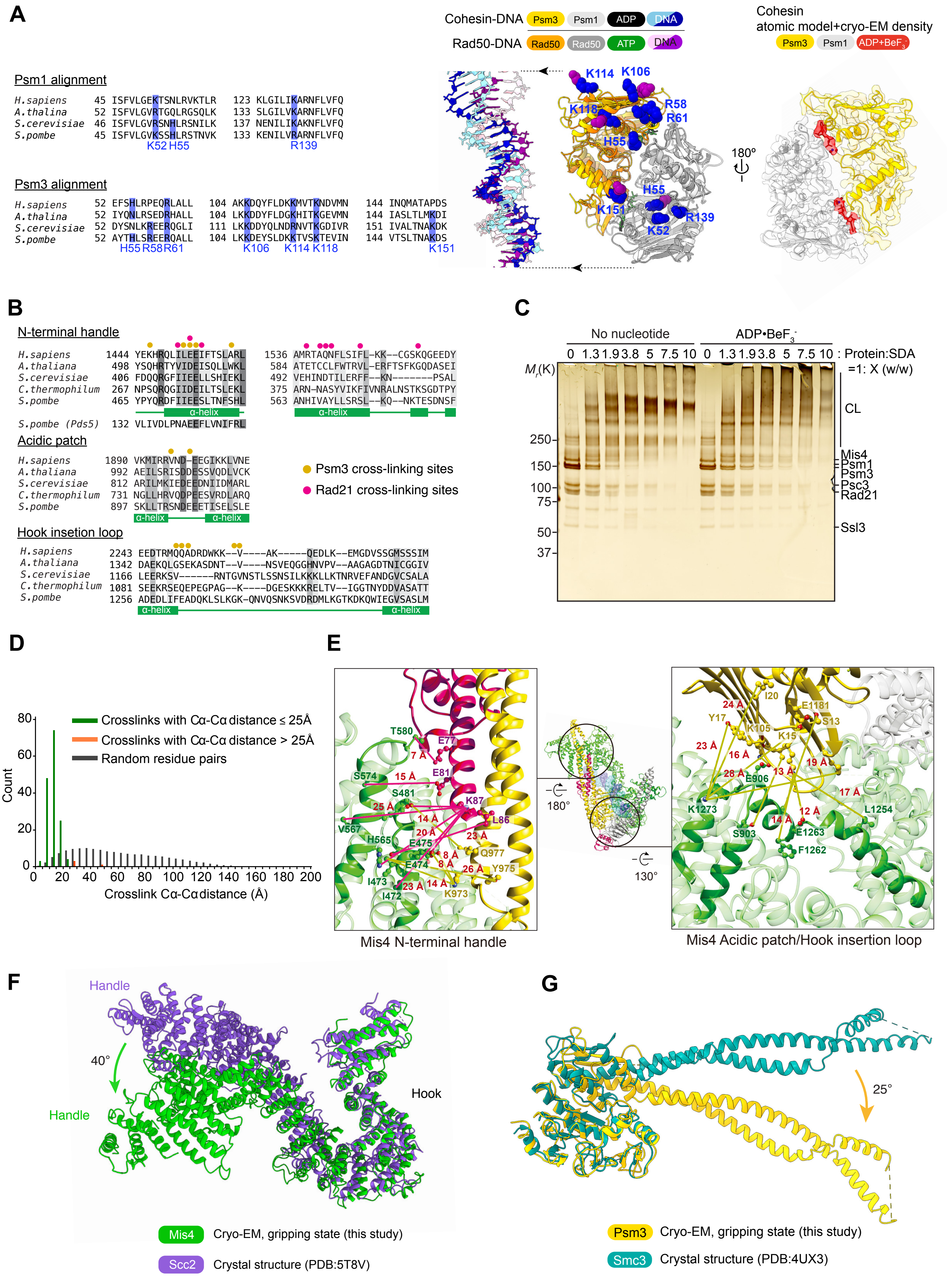

Figure S3. Analyses of the DNA Gripping State, Related to Figure 3

(A) On the left, sequence alignment of cohesin SMC heads, highlighting conserved positively charged residues that contribute to DNA contact. In the center, ATPase-head superposition of Psm3-Psm1 (gold-light grey) and *M. jannashii* Rad50 (orange-dark grey), PDB: 5F3W) (Liu et al., 2016). DNA-interacting residues are highlighted for Psm3-Psm1 (blue) and Rad50 (purple). Cohesin DNA is cyan and blue, Rad50 DNA is pink and purple. On the right, atomic model of the Psm3-Psm1 ATPase head embedded in the cryo-EM density. ADP·BeF_3^- density and model are all shown in red, apart from the beryllium atom, which is shown in blue.

(B) Sequence conservation of three Mis4 elements involved in gripping state formation. Crosslink positions identified in the CLMS experiment are highlighted. A sequence in Pds5, homologous to the Psm3 interacting motif in the Mis4 handle, is also indicated.

(C) CLMS samples of the initial binding and DNA gripping state. The SDA crosslinker was added at the indicated ratios and crosslinking induced by UV irradiation at 4 °C for 20 minutes. Samples were analyzed by SDS-PAGE followed by silver staining. Reactions at 1:1.3, 1:1.9 and 1:3.8 ratio were scaled up, combined and used for mass spectrometry analysis.

(D) Distribution of Cα-Cα distances of intra-subunit crosslinks measured in the 3D model of the cohesin gripping state. Distances within the theoretical SDA crosslinking limit (25 Å) are colored green, those >25 Å are colored in orange. A normalized distribution of Cα-Cα distances of random residue pairs within these subunits (gray) is shown as reference. Crosslinks from Mis4 are not included in this analysis due to the tendency of this subunit to self-aggregate.

(E) CLMS crosslinks between Mis4 and Psm3 (golden lines) and between Mis4 and Rad21 (red lines), mapped onto the atomic model of the gripping state. Insets contain magnified views of the Mis4 handle (left) and the Mis4 acidic patch and hook insertion loop (right).

(F) Superposition between Mis4 in the gripping state and the orthologous *C. thermophilum* Scc2 in its crystal structure conformation (PDB: 5T8V) (Kikuchi et al., 2016). Superposition of the two structures by the hook domain highlight a reconfiguration of the N-terminal handle.

(G) Comparison of Psm3 in the gripping state with *S. cerevisiae* Smc3 in its head-engaged crystal structure conformation (PDB: 4UX3) (Gligoris et al., 2014). Superposition of the two structures by their ATPase head highlight a reconfiguration of the coiled coil neck.

Supplemental Figure 4

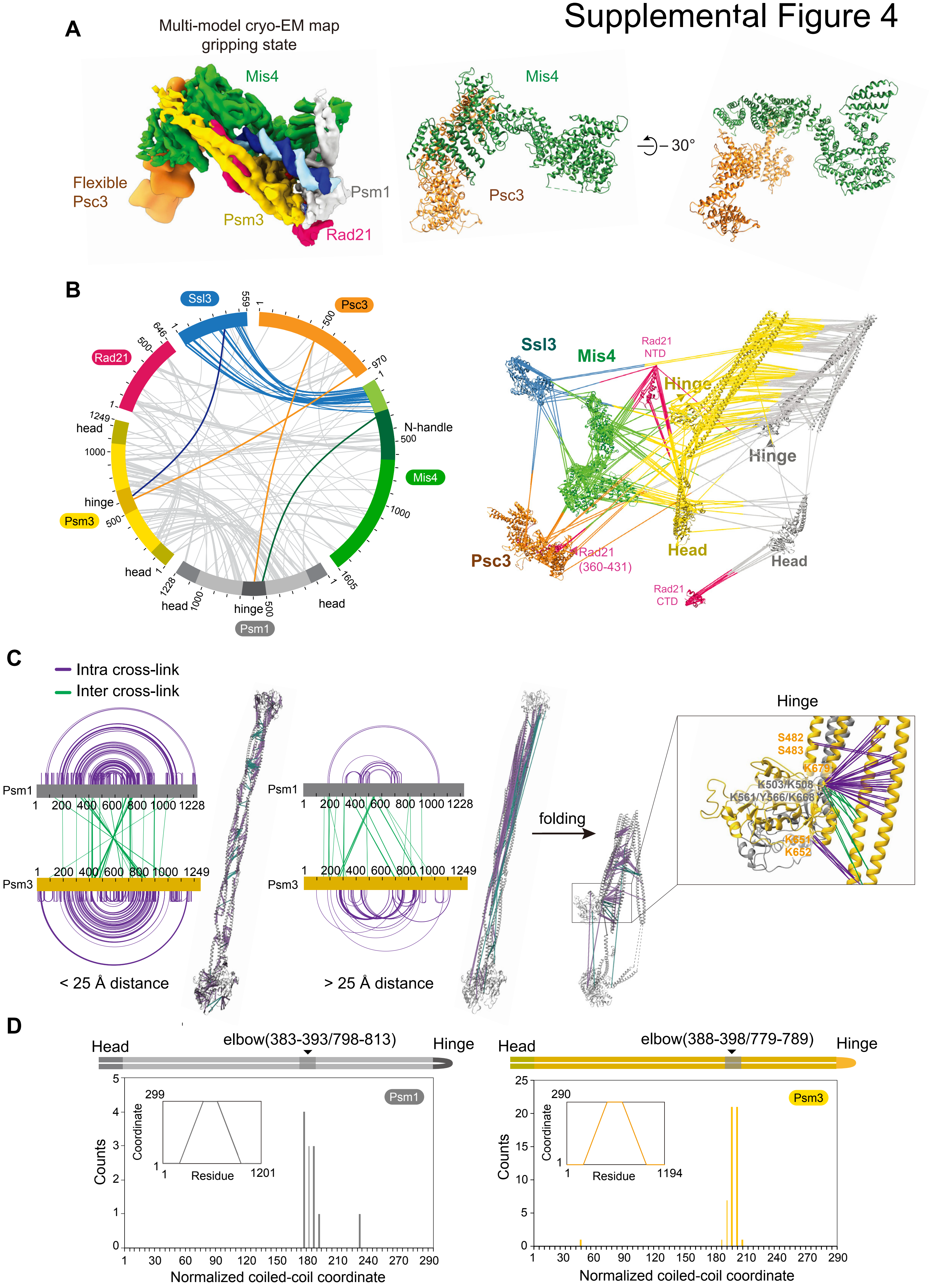

Figure S4. Supporting Analyses of the Hybrid Structural Model, Related to Figure 4

(A) Multibody refinement cryo-EM map including Psc3. On the left, result of the multibody refinement process, including the gripping state core structure and the flexibly tethered Psc3 density. On the right, atomic models of Psc3 and Mis4 in the same view, as well as rotated, to illustrate their relative orientation.

(B) Circos plot of inter-subunit interactions in the DNA gripping state. Highlighted in blue, crosslinks between Ssl3 and the Mis4 N-terminus; orange, between the Psm1-Psm3 hinge and Psc3; green, between the Psm3 hinge and the Mis4 handle; dark blue, between Ssl3 and the Psm3 hinge. The crosslinks are also displayed on an expanded atomic model of the cohesin complex.

(C) Diagrammatic and structural representation of crosslinks within (purple) and between (green) Psm1 and Psm3. An extended model of the coiled coils accommodates most crosslinks within 25 Å distance (left), however a series of crosslinks breach this distance constraint (middle). Folding the coiled coil at its predicted elbow allows many of the latter crosslinks to fall below 25 Å (right). The direction of folding is constrained by crosslinks between the hinge and coiled coil (inset).

(D) Identification of the cohesin elbow region from CLMS data. Plot of midpoints of crosslinks with residue pairs that are more than 50 amino acid apart in a normalized coiled coil coordinate system. An inset graph shows the piecewise interpolation function used to map arm residues to the unified coordinate.

Supplemental Figure 5

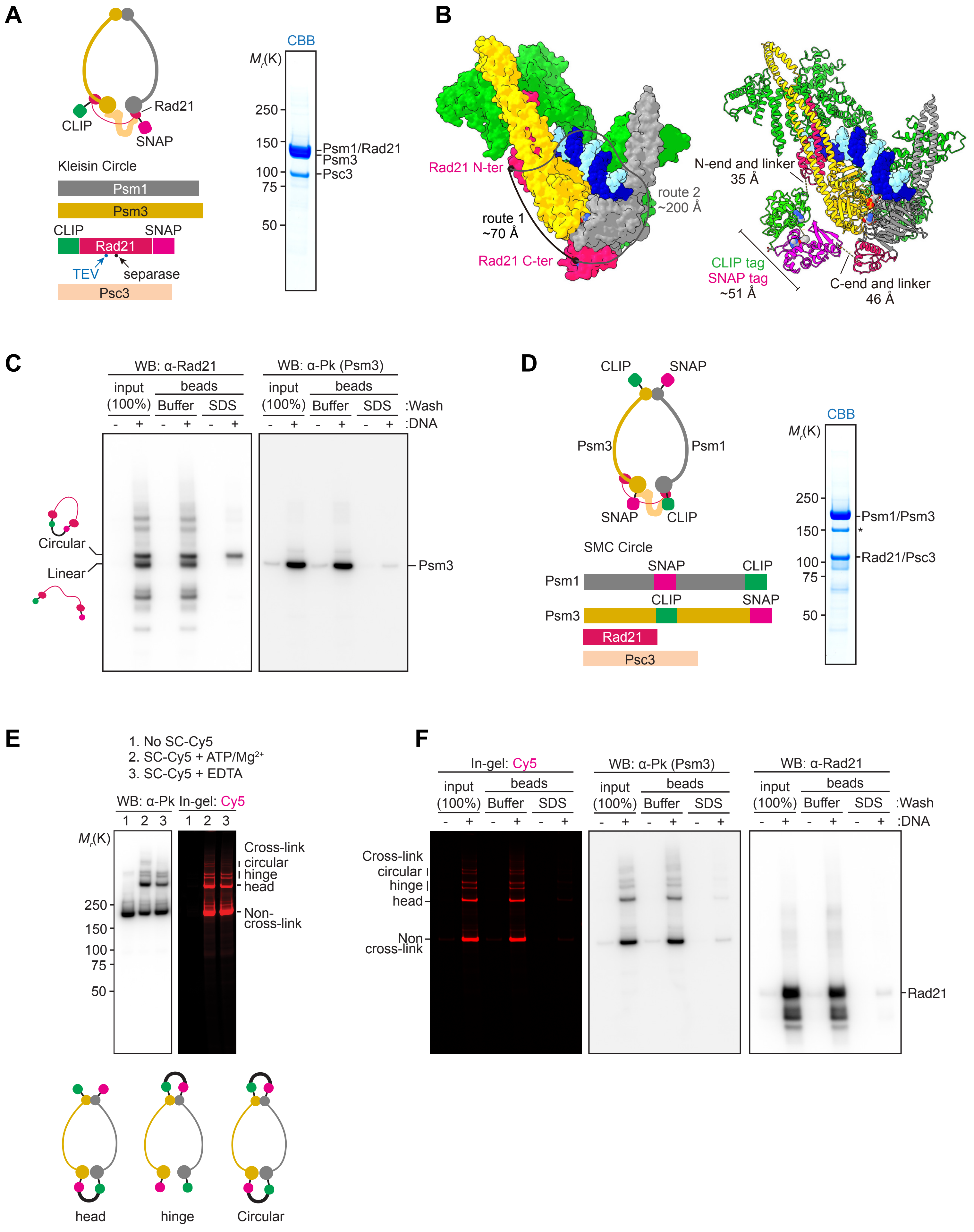

Figure S5. Supporting Information on the Kleisin Path, Related to Figure 5

(A) Schematic of the cohesin complex containing CLIP and SNAP tags at the kleisin N- and C-terminus, respectively, to circularize the kleisin. The purified complex was analyzed by SDS-PAGE and Coomassie blue (CBB) staining.

(B) Structural cartoon illustrating the kleisin circularization experiment. The SNAP and CLIP tags are represented by a structural model of the SNAP tag (PDB: 3KZZ). The length of the components is sufficient to link the kleisin N- and C-termini by the shortest route (route 1), but not via alternative routes that might entrap DNA if it was not already encircled (e.g. route 2).

(C) Immunoblot analysis of the kleisin circularization experiment in Figure 5C. DNA-bound proteins were detected using an antibody against Rad21 or an antibody against the Pk epitope fused to Psm3. Following the SDS wash, Psm3 and linear Rad21 were lost from the DNA beads.

(D) Schematic of a cohesin complex that allows SMC circularization. Psm1 has a SNAP-tag inserted between amino acids 593 and 594 at its hinge and a C-terminal CLIP tag. Psm3 in turn contains a CLIP-tag between amino acids 631 and 632 at its hinge as well as a C-terminal SNAP tag. The purified complex was analyzed by SDS-PAGE and Coomassie blue (CBB) staining.

(E) Characterization of SMC circularization. SC-Cy5 crosslinking was performed either in the presence of ATP and $MgCl_2$ (lane 2) or instead in buffer containing EDTA (lane 3). The latter should prevent head engagement, thus bands of reduced intensity likely correspond to head crosslinks. Crosslinking of both heads and hinge is expected at lower frequency than either single linkage, completing the assignment of the circularized, slowest migrating forms.

(F) In gel Cy5 detection and immunoblotting of a DNA gripping experiment using a bead-immobilized DNA loop substrate as in Figure 5D. Unlike what we observed following Rad21 circularization, SMC circularization did not result in SDS resistant protein retention on DNA.

Supplemental Figure 6

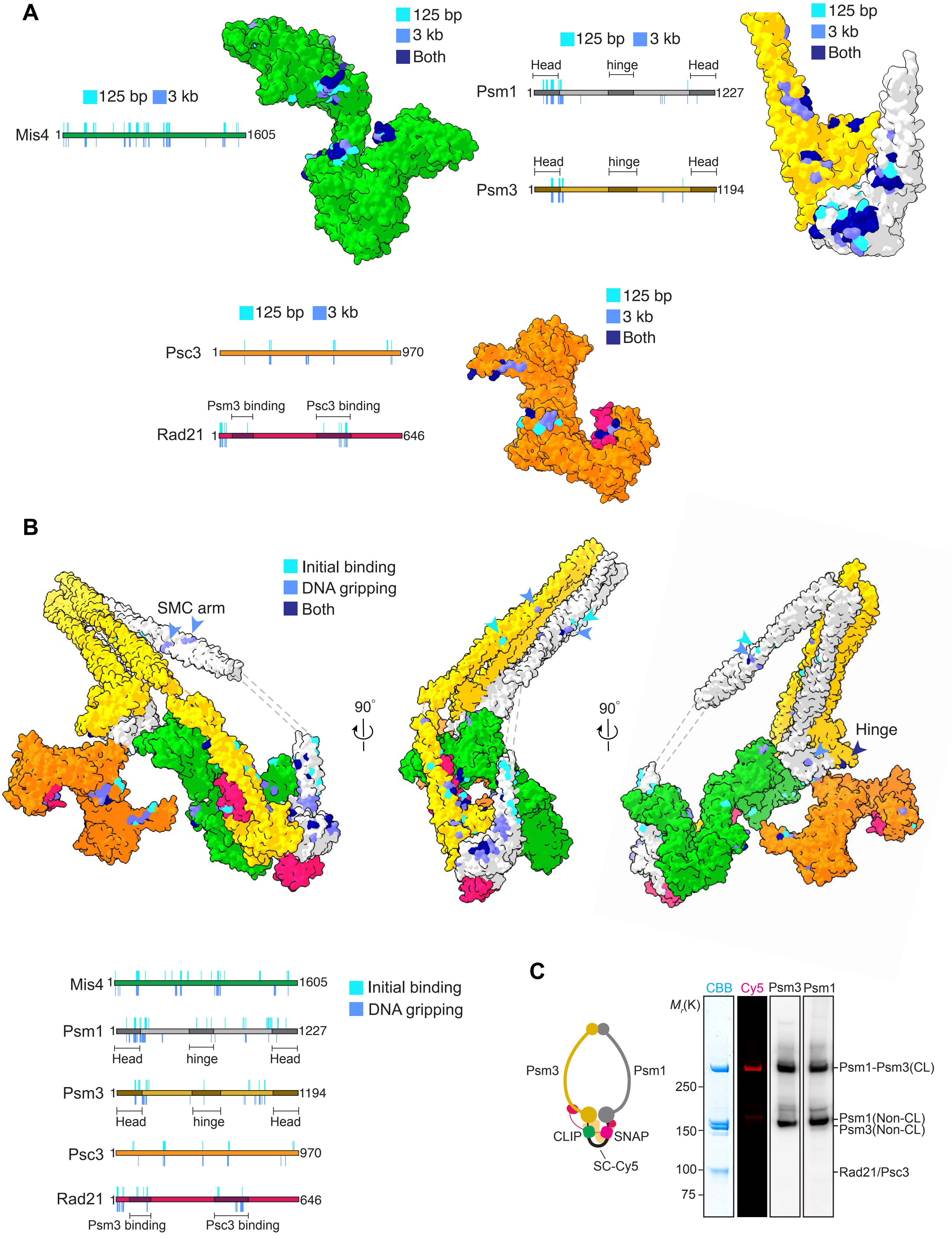

Figure S6. Additional Information on the DPC-MS Results, Related to Figure 6

(A) Schematic depiction and surface representation of individual subunits of the DNA crosslinks obtained with either a 125 bp linear DNA or a 3 kb plasmid DNA in the gripping state.

(B) Schematic depiction and surface representation on the hybrid model of the DNA crosslinks observed in the initial DNA binding mode and the gripping state. Arrowheads highlight crosslinks along the SMC coiled coils.

(C) Schematic of cohesin used for the head crosslinking experiment and analysis of the crosslink products following SC-Cy5 incubation by SDS-PAGE followed by Coomassie blue staining (CBB), in gel Cy5 detection and immunoblotting.

Supplemental Figure 7

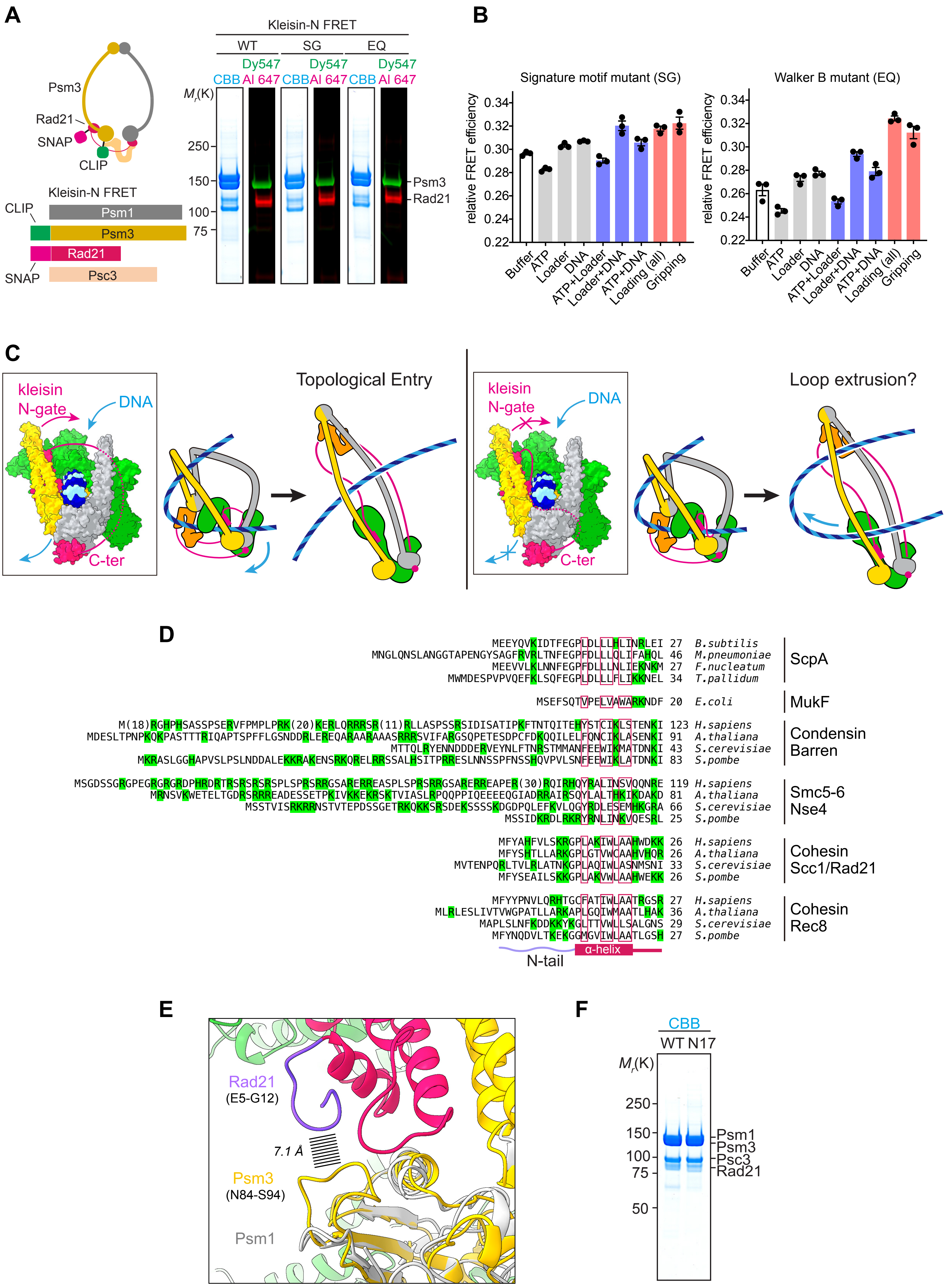

Figure S7. Further Analyses of the Kleisin N-gate and N-tail, Related to Figure 7

(A) Schematic of the kleisin N-gate FRET construct. The purified and labeled wild type (WT), signature motif (SG) or Walker B motif mutant (EQ) complexes were analyzed by SDS-PAGE followed by Coomassie blue staining (CBB) or in gel fluorescence detection.

(B) As Figure 7A, but FRET efficiencies were recorded using the signature motif and Walker B motif mutant cohesin complexes. Results from three independent repeats of the experiment, their means and standard deviations are shown.

(C) Model for the DNA fate when DNA has passed the kleisin N-gate (left), or failed to pass the kleisin N-gate (right), before reaching the ATPase heads. ATP hydrolysis and head gate opening leads to completion of DNA entry into the cohesin ring only in the first scenario. In the second, hypothetical scenario, DNA is unable to enter the cohesin ring and might instead be extruded as a DNA loop. Note that apart from kleisin binding (Haering et al., 2002; Hara et al., 2014; Li et al., 2018), Psc3's biochemically documented interaction with the cohesin complex lies at the hinge (Murayama and Uhlmann, 2015). If Psc3 retains hinge association, this would pull DNA as the SMC coiled coils extend. Under the low salt conditions typical of *in vitro* loop extrusion experiments, the cohesin loader also maintains affinity for cohesin following ATP hydrolysis (Çamdere et al., 2018). This could provide a second DNA interaction site during loop formation that changes between 'gripping' and 'slipping' states depending on the nucleotide state of the ATPase.

(D) Sequence alignment of kleisin N-tails from various SMC complexes and species. The alignment is centered on the first α-helix (boxed in red). Positively charged amino acids are highlighted green.

(E) Atomic model of the Rad21 N-tail in proximity to a Psm3 surface loop. The mean average distance between Cα atoms of the Rad21 N-tail and the Psm3 loop is indicated. Superposition of the Psm1 head is shown, that lacks a similar loop.

(F) Analysis of purified wild type (WT) and N17-cohesin by SDS-PAGE followed by Coomassie blue staining (CBB).
