# Supplementary material for: A Structure-Based Mechanism for DNA Entry into the Cohesin Ring
Source: Mol Cell. 2020 Sep 17;79(6):917–933.e9. doi: 10.1016/j.molcel.2020.07.013 (PMC7507959; doi:10.1016/j.molcel.2020.07.013)
Supplement: Table S1. Yeast Strains Used in this Study, Related to STAR Methods [file mmc2.docx]

**Table S1. Yeast Strains used in this Study, Related to the STAR Methods**

| Strain name | Species | Genotype | Purified protein |
| --- | --- | --- | --- |
| Y4483 | *S. pombe* | *pMis4-PA (LEU2), pSsl3(ura4+), h-, lue1-32, ura4-D18* | Mis4-Ssl3 |
| Y4711 | *S. pombe* | *pMis4 ΔN191-PA (LEU2), h-, lue1-32, ura4-D18* | Mis4-N191 |
| Y4443 | *S. cerevisiae* | *URA3::pGAL1-psm3-3Pk-7his / pGAL10-psm1, LEU2::pGAL1-rad21-HA-pps-protein A / pGAL10-7his-psc3, pep4Δ::HIS3* | Wild type  cohesin |
| Y4455 | *S. cerevisiae* | *URA3::pGAL1-psm3E1128Q -3Pk-7his / pGAL10-psm1E1161Q, LEU2::pGAL1-rad21-HA-pps-protein A / pGAL10-7his-psc3, pep4Δ::HIS3* | EQ-Cohesin |
| Y4755 | *S. cerevisiae* | *URA3::pGAL1-psm3K105Q,K106Q-3Pk-7his / pGAL10-psm1, LEU2::pGAL1-rad21-HA-pps-protein A / pGAL10-7his-psc3, pep4Δ::HIS3* | Psm3  K105Q/K106Q |
| Y4743 | *S. cerevisiae* | *TRP1::pGAL1-pds5-E2a-pps-proteinA, pep4Δ::HIS3* | Pds5 |
| Y6200 | *S. cerevisiae* | *URA3::pGAL1-psm3-CLIP-3Pk-7his / pGAL10-psm1-SNAP, LEU2::pGAL1-rad21-HA-pps-protein A / pGAL10-7his-psc3, pep4Δ::HIS3* | Head FRET  Wild type |
| Y6201 | *S. cerevisiae* | *URA3::pGAL1-psm3E1128Q-CLIP-3Pk-7his / pGAL10-psm1E1161Q-SNAP, LEU2::pGAL1-rad21-HA-pps-protein A / pGAL10-7his-psc3, pep4Δ::HIS3* | Head FRET  EQ-cohesin |
| Y6202 | *S. cerevisiae* | *URA3::pGAL1-CLIP-psm3-3Pk-7his / pGAL10-psm1, LEU2::pGAL1-SNAP-rad21-HA-pps-protein A / pGAL10-7his-psc3, pep4Δ::HIS3* | Kleisin-N FRET  Wild type |
| Y6203 | *S. cerevisiae* | *URA3::pGAL1-CLIP-psm3S1098R-3Pk-7his / pGAL10-psm1S1133R, LEU2::pGAL1-SNAP-rad21-HA-pps-protein A / pGAL10-7his-psc3, pep4Δ::HIS3* | Kleisin-N FRET  SG-cohesin |
| Y6204 | *S. cerevisiae* | *URA3::pGAL1-CLIP-psm3E1128Q-3Pk-7his / pGAL10-psm1E1161Q, LEU2::pGAL1-SNAP-rad21-HA-pps-protein A / pGAL10-7his-psc3, pep4Δ::HIS3* | Kleisin-N FRET  EQ-cohesin |
| Y6205 | *S. cerevisiae* | *URA3::pGAL1-psm3-3Pk-7his / pGAL10-psm1, LEU2::pGAL1-CLIP-rad21-SNAP-HA-pps-protein A / pGAL10-7his-psc3, pep4Δ::HIS3* | Kleisin circle |
| Y6206 | *S. cerevisiae* | *URA3::pGAL1-psm3hingeCLIP-headSNAP-3Pk-7his / pGAL10-psm1hingeSNAP-headCLIP, LEU2::pGAL1-rad21-HA-pps-protein A / pGAL10-7his-psc3, pep4Δ::HIS3* | SMC circle |
| Y6207 | *S. cerevisiae* | *URA3::pGAL1-psm3-3Pk-7his / pGAL10-psm1, LEU2::pGAL1-rad21ΔN17-HA-pps-protein A / pGAL10-7his-psc3, pep4Δ::HIS3* | N17-cohesin |

All budding yeast strains are W303 background (*ura3-52, ade2-1, trp1-1, can1-100, leu2-3,112, his3-11,15*)

PA: Protein A, 3Pk: 3xV5 tag, 7his: 7x histidine tag, pps: PreScission protease site, HA: HA tag
