# Supplementary material for: A Structure-Based Mechanism for DNA Entry into the Cohesin Ring
Source: Mol Cell. 2020 Sep 17;79(6):917–933.e9. doi: 10.1016/j.molcel.2020.07.013 (PMC7507959; doi:10.1016/j.molcel.2020.07.013)

Table S2. Image Processing Workflow for the Cryo-EM Core Structure, as well as Multibody Refinement Workflow that Led to the Identification of a Separate Rigid Body Identified as Psc3, Related to the STAR Methods

Cohesin Core Structure Image Processing Workflow

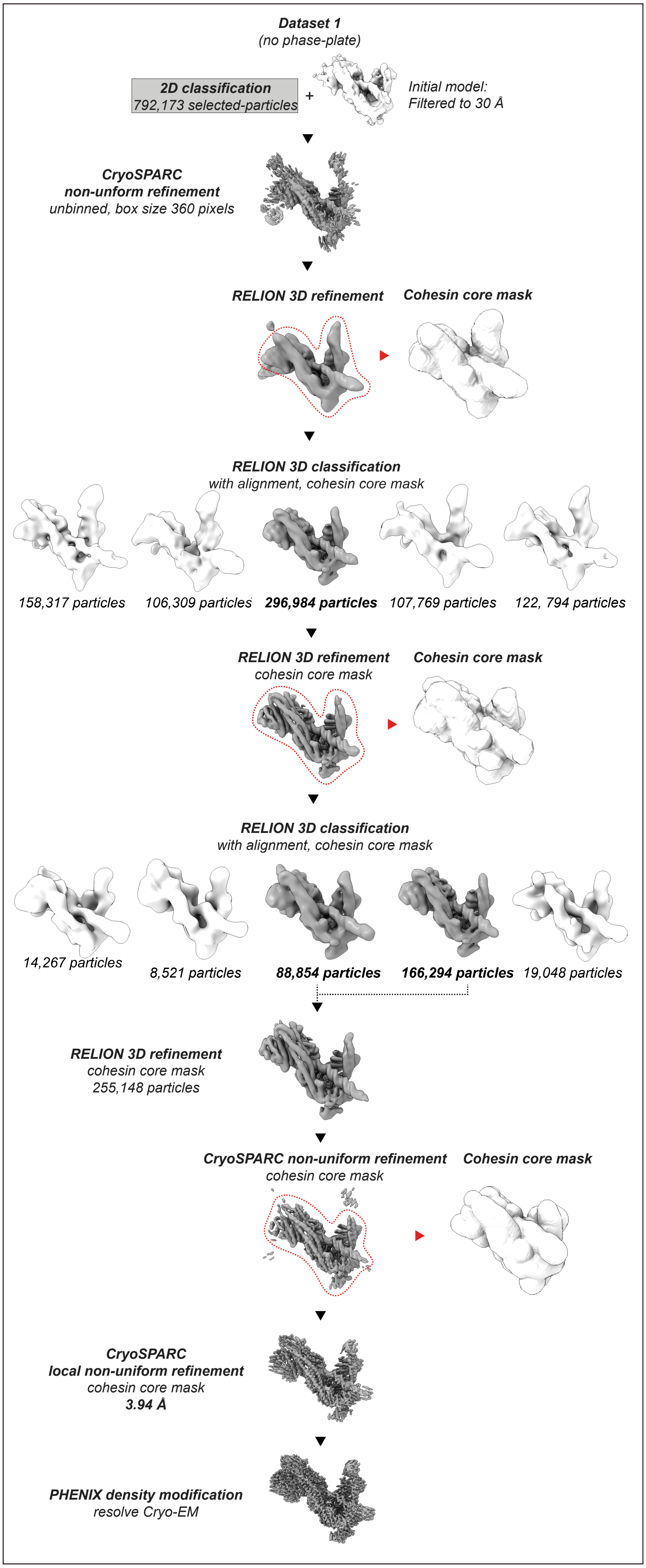

Multibody Refinement Workflow

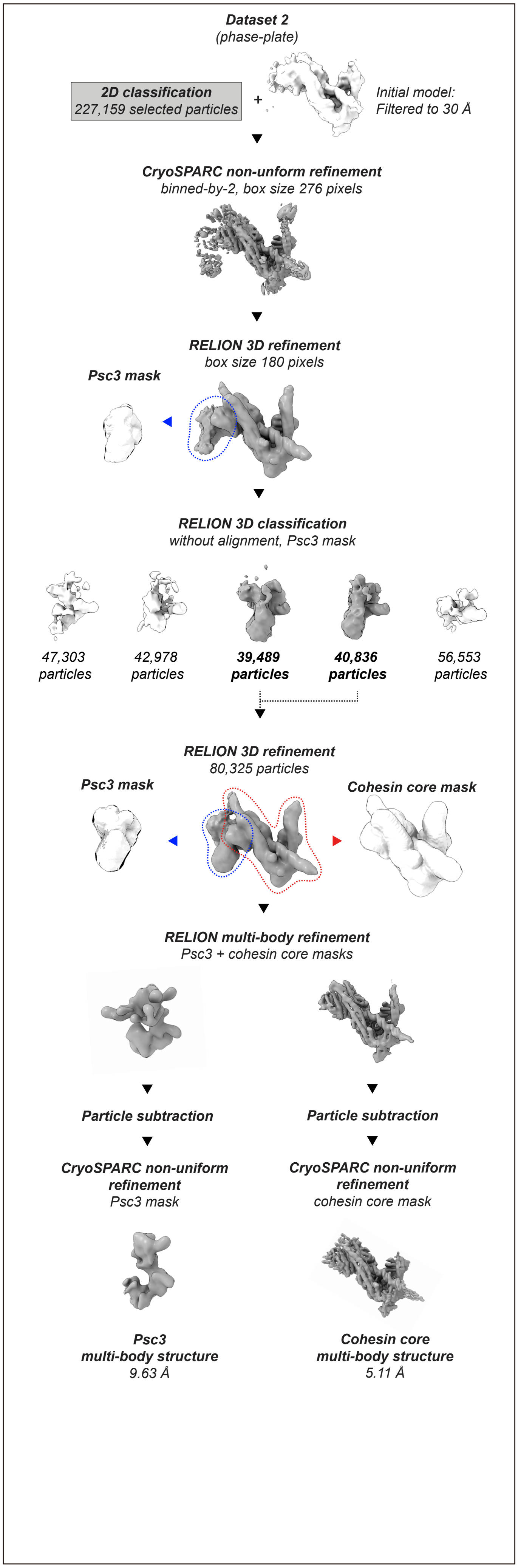

Supplement: Table S2. Image Processing Workflow for the Cryo-EM Core Structure as well as the Multibody Refinement Workflow that Led to Identification of a Separate Rigid Body Identified as Psc3, Related to STAR Methods [file mmc3.pdf]
