# Supplementary material for: A Structure-Based Mechanism for DNA Entry into the Cohesin Ring
Source: Mol Cell. 2020 Sep 17;79(6):917–933.e9. doi: 10.1016/j.molcel.2020.07.013 (PMC7507959; doi:10.1016/j.molcel.2020.07.013)
Supplement: Table S3. Cryo-EM Data Collection, Refinement, and Validation Statistics, Related to STAR Methods [file mmc4.docx]

**Table S3. Cryo-EM Data Collection, Refinement and Validation Statistics, Related to the STAR Methods**

|  | Cohesin-DNA-Loader Gripping State |
| --- | --- |
| Data collection and processing |  |
| Magnification | 75,000 |
| Voltage (kV) | 300 |
| Electron exposure (e^–^/A^2^) | 33.8 |
| Defocus range (µm) | -2.0 to -4.0 |
| Pixel size (Å) | 1.09 |
| Symmetry imposed | C1 |
| Initial particle images (no.) | 883,184 |
| Final particle images (no.) | 255,148 |
| Map resolution (Å) | 3.94 |
| FSC threshold | 0.143 |
| Map resolution range (Å) | 3.5 to 6 |
| Refinement* |  |
| Model resolution (Å) | 4.39 |
| FSC threshold | 0.5 |
| Map sharpening B factor (Å2) | -199 |
| Model composition |  |
| Non-hydrogen atoms | 19,536 |
| Protein residues | 2,264 |
| Nucleotides | 64 |
| Ligands | 4 |
| Validation |  |
| MolProbity score | 2.07 |
| Clashscore | 10.83 |
| Poor rotamers (%) | 0.26 |
| Ramachandran plot |  |
| Favored (%) | 91.01 |
| Allowed (%) | 8.99 |
| Disallowed (%) | 0 |

* The statistical analysis excludes residues 552 to 583 from Rad21 and residues 209 to 302 from Mis4 that were built by homology modelling and rigid body fitting into the cryo-EM map, but not refined using Phenix real space refinement.
